# Patient-derived follicular lymphoma spheroids recapitulate lymph node signaling and immune profile uncovering galectin-9 as a novel immunotherapeutic target

**DOI:** 10.1038/s41408-024-01041-7

**Published:** 2024-05-02

**Authors:** Cèlia Dobaño-López, Juan García Valero, Ferran Araujo-Ayala, Ferran Nadeu, Fabien Gava, Carla Faria, Marine Norlund, Renaud Morin, Pascale Bernes-Lasserre, Fabian Arenas, Marta Grau, Cristina López, Irene López-Oreja, Neus Serrat, Ares Martínez-Farran, Lluís Hernández, Heribert Playa-Albinyana, Rubén Giménez, Silvia Beà, Elías Campo, Jean-Michel Lagarde, Armando López-Guillermo, Laura Magnano, Dolors Colomer, Christine Bezombes, Patricia Pérez-Galán

**Affiliations:** 1 Fundació de Recerca Clínic Barcelona - Institut d’Investigacions Biomèdiques August Pi i Sunyer, Barcelona, Spain; 2 Centro de Investigación Biomédica en Red-Oncología (CIBERONC), Madrid, Spain; 3 Université de Toulouse, INSERM, CNRS, Université de Toulouse III-Paul Sabatier, Centre de Recherches en Cancérologie de Toulouse, Toulouse, France; 4 IMACTIV-3D, Toulouse, France; 5 University of Barcelona, Medical School, Barcelona, Spain; 6 Secció Hematopatologia, Servei d’Anatomia Patològica, Hospital Clínic, Barcelona, Spain; 7 Servei Hematologia, Hospital Clínic, Barcelona, Spain

**Keywords:** B-cell lymphoma, Cancer immunotherapy

## Abstract

Follicular lymphoma (FL), the most common indolent non-Hodgkin lymphoma, constitutes a paradigm of immune tumor microenvironment (TME) contribution to disease onset, progression, and heterogenous clinical outcome. Here we present the first FL-Patient Derived Lymphoma Spheroid (FL-PDLS), including fundamental immune actors and features of TME in FL lymph nodes (LNs). FL-PDLS is organized in disc-shaped 3D structures composed of proliferating B and T cells, together with macrophages with an intermediate M1/M2 phenotype. FL-PDLS recapitulates the most relevant B-cell transcriptional pathways present in FL-LN (proliferation, epigenetic regulation, mTOR, adaptive immune system, among others). The T cell compartment in the FL-PDLS preserves CD4 subsets (follicular helper, regulatory, and follicular regulatory), also encompassing the spectrum of activation/exhaustion phenotypes in CD4 and CD8 populations. Moreover, this system is suitable for chemo and immunotherapy testing, recapitulating results obtained in the clinic. FL-PDLS allowed uncovering that soluble galectin-9 limits rituximab, rituximab, plus nivolumab/TIM-3 antitumoral activities. Blocking galectin-9 improves rituximab efficacy, highlighting galectin-9 as a novel immunotherapeutic target in FL. In conclusion, FL-PDLS maintains the crosstalk between malignant B cells and the immune LN-TME and constitutes a robust and multiplexed pre-clinical tool to perform drug screening in a patient-derived system, advancing toward personalized therapeutic approaches.

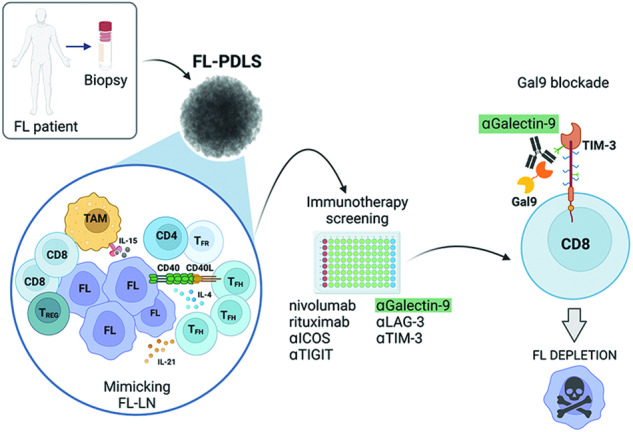

## Introduction

Follicular Lymphoma (FL) is the most common indolent non-Hodgkin’s lymphoma (NHL) and is considered a chronic and incurable disease [1]. Despite the high response rates to R-CHOP induction therapy, relapses are a frequent event. The risk of histologic transformation to diffuse large B cell lymphoma (DLBCL) increases over time (2–3% per year) [2], and confers a dismal prognosis [3]. FL arises from the malignant transformation of germinal center (GC) B cells that most frequently (∿85%) acquire the t(14;18)(q32;q21) translocation, leading to BCL2 overexpression [4]. In addition, genetic alterations in histone-modifying enzymes are recognized as a central hallmark of FL, being *KMT2D*, *CREBBP*, and *EZH2* [5] the most frequently mutated genes, together with other relevant lesions participating in pathogenic processes (i.e., *MEF2B*, *HVEM*, and *RRAGC*) [6–8]. Moreover, these alterations induce a reshaping in the immune tumor microenvironment (TME), favoring the generation of this disease and its progression [9, 10].

FL represents a paradigm of dependence on the TME [11–13], and current stratification scores include both genetic alterations and immune signatures [14–16]. This TME is composed of an intricate network of cytokines and immune modulators expressed by non-malignant cells present in the normal GC structures of lymph nodes (LN). The main players that support FL cells and maintain the GC structure are CD4^+^ T follicular helpers (T_FH_) and follicular dendritic cells (FDC), which by means of CD40L, IL-4, and IL-21 cytokine signaling, contribute to tumor survival and proliferation [17–19]. Moreover, FL cells are involved in the recruitment of CD4^+^ T regulatory cells (T_REG_), in charge of inhibiting anti-tumor immune responses, hampering CD8^+^ granzyme B and perforin release [20]. In addition, these CD8^+^ exhibit an exhaustion profile with high levels of PD-1 and TIM-3 [21]. Nevertheless, the myeloid compartment trans-presents IL-15 which will stimulate NK and CD8^+^ cell activity. Likewise, TAMs encompass several subpopulations with opposing roles in the TME. Therefore, conflicting prognosis values were obtained using the classical TAM marker CD163 [22]. In this regard, we have demonstrated that the percentage of CSF-1R^+^ macrophages (Mϕ) correlates with FL grade and risk of transformation to an aggressive lymphoma [23].

In order to study this complex and heterogeneous network, patient-derived systems that recapitulate microenvironment cues are mandatory. FL cell lines are not representative of FL pathology or its heterogeneity, and patient-derived xenograft (PDX) models fail to recapitulate the human microenvironment. In the last years, there has been an evolution toward patient-derived 3D cultures and organoids in many solid tumors [24], yet lymphomas are scarce [25, 26] and with limited TME [27]. Therefore, there is a need to establish an amenable system recapitulating the main pathogenic pathways delivered to the tumor B-cell in the LN, together with the immune activation/suppression status of T cells and the myeloid compartment. This system would be instrumental in the context of precision medicine. In the same line, we have recently developed a mantle cell lymphoma (MCL) patient-derived 3D model co-culturing MCL primary cells with autologous T cells and monocytes from healthy donors [28].

Here we present the first patient-derived lymphoma spheroids (PDLS) from FL samples (FL-PDLS) that recapitulate the signaling cues of the tumor niche in the GC together with monocytes and autologous T cells, building a system for immunotherapies and cell therapies intervention. In this context, we have uncovered a role for galectin-9 in T-cell responses that may deserve attention in FL immunotherapeutic approaches.

## Methods

### FL-PDLS generation

**Fig. 1:**
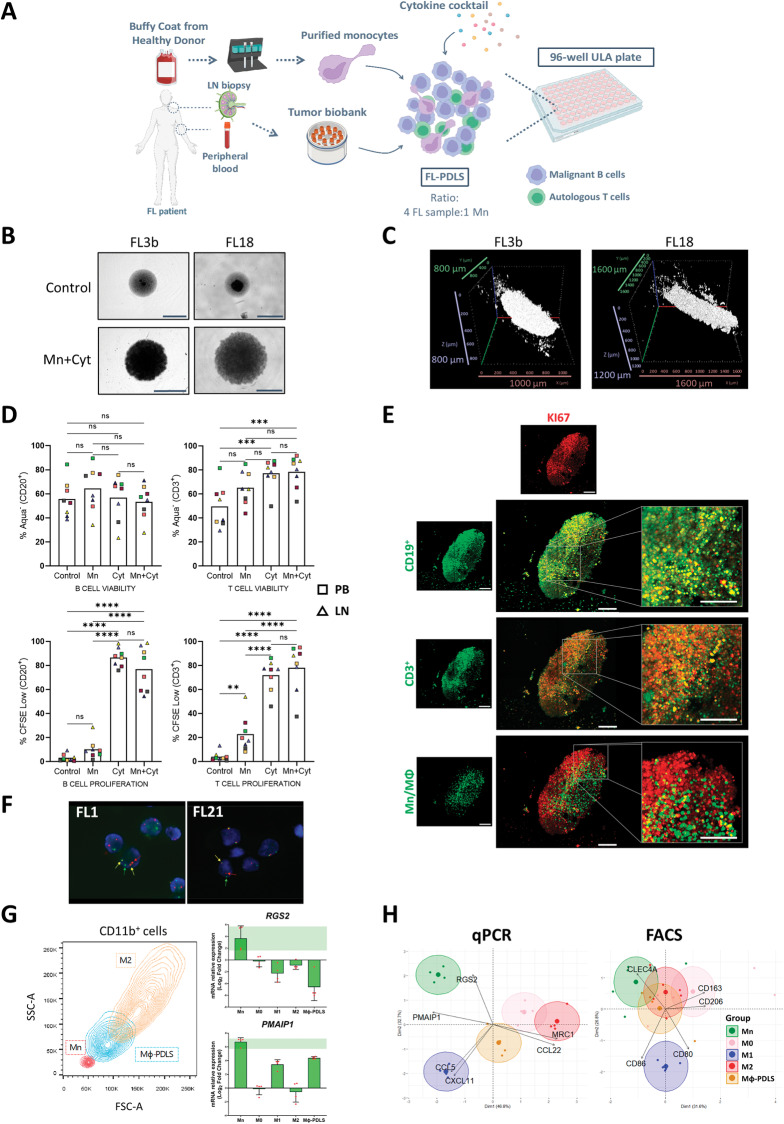
FL-PDLS is a novel patient-derived model including an immune microenvironment. **A** Schematic representation of the workflow for FL-PDLS generation. Created with BioRender.com. **B** Brightfield images (Cytation 1) of two representative cases after 7 days of culture showing non-stimulated PDLS (Control) and the PDLS medium with allogeneic monocytes (Mn+Cyt). Magnification 4× and 1000 µm scale. **C** 3D structure obtained by SPIM microscopy of PDLS shown in (**B**), under complete condition (Mn+Cyt). **D** CD20^+^ and CD3^+^ population viability (upper panel) was determined by the percentage of Aqua^-^ flow cytometry staining, and proliferation (lower panel) was measured by the percentage of CFSE low signal after 7 days of culture in the four experimental scenarios. Patient coding is included in Table 1. One-way ANOVA followed by the Holm–Sidak post hoc test was applied. **E** FL-PDLS immunofluorescence of CD19^+^, CD3^+^, or Mn/Mϕ cells (green), merged with Ki-67^+^ (red) to determine the proliferation of each population by signal colocalization (yellow) at day 7 of culture. Captured in confocal Leica TCS SPE microscope. Scale 200 µm. **F** *BCL-2/IGH* rearrangement by FISH using *IGH/BCL2* dual fusion dual color in FL1 and break-apart probe in the case FL21. FL1: the signal constellation illustrates two yellow signals (yellow arrow) corresponding to the *IGH*/*BCL2* fusion and one red (red arrow), and one green signal (green arrow) for the unrearranged *BCL2* and *IGH*, respectively. Normal cells have two green signals and two red signals. FL21: the signal constellation shows a yellow signal (yellow arrow) for the unrearranged *BCL2* and green and red signals for the rearranged *BCL2* allele. Normal cells display two yellow signals. **G** Side scatter (SSC-A) vs forward scatter (FSC-A) plot of CD11b^+^ cells from Mn, from day 6 Mϕ-PDLS, and from M2 macrophages (left). Gene expression of Mn makers (RGS2 and PMAIP1) by RT-qPCR in CD11b^+^ cells sorted from day 7-FL-PDLS, compared to Mn, M0, M1, or M2 macrophages. Values are relative to M0 macrophages (mean, *n* = 4) (right). **H** PCA diagrams clustering Mɸ-PDLS with in vitro differentiated M0, M1, M2 and non-differentiated Mn, based on CXCL11, CCL5, MRC1, CCL22, RGS2 and PMAIP1 gene expression levels measured by RT-PCR (left), or based on flow cytometry expression of CD163, CD206, CLEC4A, CD80, and CD86 (right).

FL samples from LN or peripheral blood (PB) (*n* = 20) (Table 1) were thawed in sterile conditions, resuspended in enriched medium [29] (RPMI Glutamax (Gibco, Thermo Fisher Scientific, Waltham, MA, USA); 15% FBS (Gibco, Thermo Fisher Scientific); 1,1% ITS (Sigma-Aldrich, St. Louis, MO, USA); 1% HEPES (Sigma-Aldrich); 1% Pyruvate (Gibco, Thermo Fisher Scientific); 1% Non-Essential AA (Gibco, Thermo Fisher Scientific); 0.1% 2β-mercaptoethanol (Sigma-Aldrich); 0.5% Gentamicine (Sigma-Aldrich)), and counted using Neubauer chamber system with trypan blue to assess initial cell viability. To assess proliferation, cells were labeled with 0.5 µM carboxyfluorescein succinimidyl ester (CFSE) cell tracker (Thermo Fisher Scientific) following the manufacturer’s instructions. CFSE-labeled FL samples were then mixed with monocytes (isolation described in supplemental methods) at 4:1 ratio (FL:monocytes), seeding 50,000 cells/well and 12,500 monocytes/well in a final volume of 200 µL/well in Nunclon^TM^ Sphera^TM^ 96-wells Ultra-Low Attachment (ULA) microplates (Thermo Fisher Scientific) in the enriched medium mentioned above, supplemented with the following cytokines (PDLS medium): 50 ng/mL CD40L-HA tagged (R&D Systems, Minneapolis, MN, USA), 1 µg/mL anti-HA-Tag antibody (R&D Systems), 100 ng/mL IL-21 (Peprotech, Cranbury, NJ, USA), 10 ng/mL IL-4 (Peprotech) and 50 ng/mL IL-15 (R&D Systems). PDLS were maintained at 37 °C 5% CO_2_ for up to 10 days. The workflow for FL-PDLS generation is detailed in Fig. 1A.

**Table 1:** FL patient characteristics.

Study label	Sex/age^a^	Sample type^b^	Disease status^c^	Histological Grade^d^	Stage^e^	FLIPI^f^	Treatments^g^	Response to 1st tt^h^	POD24^i^	Cell count Lympho B (10^9^/L)	% Lympho B^j^	Code
FL1	F/75	PB	D	2	IVA	H	R-COP/R-mnt	PR	N	76.46	78	
FL2	F/52	LN	D	1	IV	H	R-CHOP/R-mnt	CR	N	3.13	85	
							R-ESHAP					
							ASCT					
							R-GEMOX					
							lfosfamide					
							Rx					
							CAR-T (axicel)					
FL3a	M/63	PB	Pt	2	IV	M	R-CHOP/R-mnt	CR	N	190.77	95	
							GA-Benda + ASCT					
							Parsaclisib + Ibru					
FL3b	M/63	PB	Pt	2	IV	M	R-CHOP/R-mnt	CR	N	78.06	83	
							GA-Benda + ASCT					
							Parsaclisib + Ibru					
FL4	M/27	PB	D	2	IV	M	R-CHOP	CR	N	13.75	75	
FL7	M/65	LN	R3	2	IV	H	CHOP	PR	Y	17.58	72	
							FCM					
							R-COP					
FL9	M/75	PB	D	1	IV	M	W&W	–	–	11.69	78	
FL11	M/37	LN	R1	N/A	N/A	N/A	Chemo-Rx	N/A	N	N/A	57	
							R					
FL12	M/35	LN and PB	D	2	IV	H	R-CHOP/R-mnt	CR	N	1.86	60	
							R-Benda/R-mnt					
FL14	M/43	LN and PB	D and R1	1	IV	L	CHOP	SD	Y	14.04	80	
							ESHAP					
							FCM + ASCT					
							Burkimab					
FL16a	F/51	LN and PB	D	2	IV	H	R-CHOP/R-mnt	CR	N	1.043	64	
FL16b	F/51	PB	D	2	IV	H	R-CHOP/R-mnt	CR	N	9.64	70	
FL18	M/56	LN	Pt	2	I	N/A	Rx	CR	N	N/A	55	
FL21	F/58	PB	R2	1	IV	M	GA-CHOP/GA-mnt	CR	Y	30.75	78	
							R-Benda					
							R2					
FL23	M/55	Ovary	D	2	IV	M	R-CHOP/R-mnt	CR	N	0.79	40	
FL25	M/32	LN	R1	3A	I	N/A	Rx	CR	N	0.7	40	
							R+Rx					
FL29	M/72	PB	R1	2	IV	H	R-COP	CR	Y	2.31	68	
							FCM/PDN					
FL31	M/58	LN	R1	1	IV	N/A	Rx	CR	N	1.36	80	
FL32	F	PB	N/A	N/A	N/A	N/A	N/A	N/A	N/A	N/A	92	
FL34	M/59	PB	R1	3A	IV	H	R-CHOP/R-mnt	CR	N	209.21	94	
							R-Benda					

^a^*F* Female; *M* Male.

^b^*PB* Peripheral Blood, *LN* Lymph Node.

^c^Samples were obtained at *D* diagnosis, *R* relapse, *Pt* Pretreatment, *NA* Not Available.

^d^Evaluated by two independent pathologists.

^e^*Ann* Arbor stage.

^f^*FLIPI* Follicular Lymphoma International Prognostic Index (High (H):≧3, *Medium* (M), 2; Low (L):0–1).

^g^All treatments; *R* Rituximab, *R-mnt* Maintenance Rituximab, *RTx* Radiotherapy, *GA* Obinutuzumab, *Benda* Bendamustine, *CHOP* Chemotherapy combination of Cyclophosphamide Hydroxydaunorubicin. Oncovin and Prednisone, *ASCT* Autologous Stem Cell Transplantation, *Ibru* Ibrutinib, *FCM* Chemotherapy combination of Fludarabine, Cyclophosphamide and Mitoxantrone, W&W Watch and Wait, *CFM* Chemotherapy combination of Cyclophosphamide cyclophosphamide, methotrexate and 5 fluorouracil, *PDN* Prednisone,

^h^*tt* treatment, *CR* Complete Response, *PR* Partial Response, *SD* Stable Disease.

^i^*POD24* Progression of disease within 2 years.

^j^from B-cell panel (CD19, CD20, CD22 and CD79b) in routine diagnosis. *Y* Yes; *N* No; *N/A* Not available.

### Drug assays and activation analysis

Six FL-PDLS replicates per experimental condition were cultured in 150 µL/well of PDLS medium + Mn and treated after 72 h (day 3) with 50 µL/well containing the drugs diluted in PDLS medium. The following antibodies and doses were used: 3.75 µg/mL rituximab (provided by Hospital Clínic pharmacy), 10 µg/mL anti-ICOS, 5 µg/mL anti-TIM-3, 2 µg/mL anti-LAG-3, and 10 µg/mL anti-TIGIT, all functional grade antibodies from Thermo Fisher Scientific, 10 µg/mL nivolumab (Selleck Chemicals LLC, Houston, TX, USA) and 2.5 µg/mL anti-galectin9 (Biolegend, San Diego, CA, USA). CHOP components, provided by the Hospital Clínic pharmacy, were used at the following concentrations: cyclophosphamide (0.1 µg/mL), doxorubicin hydrochloride (0.5 µg/mL), vincristine sulfate (0.014 µg/mL) and prednisone (1 µg/mL). After 72 h of treatment (day 6), PDLS were mechanically disaggregated to analyze cell viability (LIVE/DEAD Fixable Aqua, Thermo Fisher Scientific) and cell population proportions (CD20, CD3, CD4, CD8) by flow cytometry (BD LSRFortessa SORP-HTS, BD Biosciences, Franklin Lanes, NJ, USA). To be able to determine cell absolute number of viable cells, disaggregated PDLS were analyzed by reading a fixed volume using a High Throughput Sampler (HTS) integrated in the cytometer. To quantify IFNɣ levels after 72 h of treatment, PDLS supernatants were pooled and stored at −80 °C. Cytometric Bead Array (CBA) kit was then used following the manufacturer’s instructions (BD Biosciences). Data was analyzed using FCAP Array^TM^ v.3.0 Software (BD Biosciences).

Additional materials and methods, including FL-PDLS imaging, immune profile, immunofluorescence, RNAseq, and metadata analysis, together with a complete list of antibodies (Table S1), are provided as Supplementary Information.

## Results

### FL-PDLS generation and characterization

To recreate an ex vivo FL model that recapitulates immune TME signaling in the LN, composed of non-malignant immune cells such as CD4 T_FH_, T follicular regulatory cells (T_FR_), and T_REG_ together with CD8 cytotoxic cells and TAMs [13, 30], we have established a 3D model using primary FL samples (*n* = 20, Table 1) composed by malignant B FL cells and autologous T cells, together with monocytes (Mn) (1Mn:4FL ratio) purified from healthy donors. We optimized a PDLS medium containing the following cytokines to recapitulate the FL–LN niche: CD40L, IL-21, IL-4, and IL-15 in an enriched medium (described in materials and methods). Several cytokine combinations were tested that maintained B and T cell viability (Figure S1), CD40L clustering and IL-21 were fundamental for B-cell proliferation, while the addition of IL-15, trans-presented by monocytes, promoted T cell proliferation [31].

Either cryopreserved LN biopsies or PB samples were seeded as a multicellular suspension in ULA plates, as shown in the workflow (Fig. 1A), to facilitate cell aggregation and growth. PDLS clustering occurred within the first 12 h (Supplemental Video 1), successfully maintaining a rounded structure for up to 7–10 days of culture, containing darker dense proliferation cores where CFSE staining disappeared (Figs. 1B and S2A). To characterize the real 3D structure, PDLS were imaged by Selective Plane Illumination Microscopy (SPIM, ZEISS Lightsheet Z.7, Imactiv 3D, Toulouse, France), showing self-organized disc-shaped structures with a mean volume of 1.84 mm^3^ (Fig. 1C and Supplemental Video 2). We then analyzed the viability and proliferation of B and T cells in the PDLS. The culture without cytokines (control) maintained B cell viability (mean: 53.68), and the monocytes moderately improved this effect (mean: 64.45) (Fig. 1D). Cell proliferation, evaluated by accumulative CFSE loss, was prominently engaged when cytokines were added (PDLS medium, Cyt) in B and T cells (mean 86.78 and 71.9 respectively), whereas the co-culture with monocytes alone (Mn) just induced significant proliferation in T cells (mean: 22.68) while the effect on B cells was minor. Likewise, both monocyte co-culture (Mn) and PDLS medium (Cyt) alone enhanced T cell viability (mean: 65.26 and 77.23 respectively), reaching a mean percentage above 78% when both were combined (Fig. 1D). Thus, we decided to combine Cyt (PDLS medium) and Mn to better mimic FL-TME signaling and interactions including the presence of the myeloid compartment. Proliferation was further confirmed by confocal microscopy labeling Ki-67 in CD19^+^, CD3^+^, and macrophages (Fig. 1E). The colocalized signals in yellow validate the expression of Ki67 in B and T cells, whereas in monocytes/macrophages, no double signaling was detected, confirming the low proliferation capacity of human monocytes cultured in vitro [32]. B, T cell and macrophage distribution in the PDLS are shown in Fig. S2B. The cellular composition was evaluated in day 7-PDLS, displaying a mean of 60.11% CD20^+^, 4.62% CD11b^+^, and 35.27% CD3^+^, where CD4^+^/CD8^+^ account for 44%/55% on average (Fig. S2C). Importantly, FL-PDLS maintained the FL hallmark *BCL2* rearrangement as demonstrated by fluorescence in situ hybridization (Fig. 1F). Likewise, we analyzed light chain restriction in the initial sample and in day 6-PDLS (Fig. S2D), demonstrating that most of B cells maintained the original isotype, with a small portion of cells with a different isotype, indicating some proliferation of existing normal cells in the initial sample. Moreover, tumor cells (CD20 + CD10+ kappa+, in red) were CD43 dim and CD305- at day 0, and this specific FL markers [33] are preserved in the PDLS (shown for FL1, Fig. S2D).

We next analyzed the evolution of monocytes introduced in the FL-PDLS. As shown in Fig. 1G, CD11b^+^ cells in the day, 6-PDLS increased size (FSC-A) and complexity (SSC-A) when compared with day 0 CD11b^+^ Mn and downregulated the expression of the monocyte genes *RGS2* and *PMAIP1* [34] indicating their differentiation toward macrophages in the FL-PDLS system. We next characterized their phenotype by qPCR (*CXCL11/CCL5* for M1 and *MRC1/CCL22* for M2) and by flow cytometry (CD80/CD86 for M1 and CD163/CD206/CLEC4A for M2) [34]. Most of the M1 and M2-like markers increase with the PDLS condition (Figs. 1H and S2E), indicating that monocytes added in the PDLS culture, after 7 days, partially differentiate into macrophages and polarize within an intermediate state between an activated (M1) and a tumor supportive (M2) phenotype. This phenomenon is clearly displayed in the corresponding PCA diagrams for qPCR and flow cytometry protein markers (Fig. 1H).

In summary, FL-PDLS is organized in a disc-shaped 3D structure composed of proliferating and viable B tumor cells and autologous T cells, together with macrophages with an intermediate M1/M2 phenotype.

### FL-PDLS from PB samples recapitulate LN signaling pathways

To determine if FL-PDLS engages a transcriptional program close to that of LN-resident FL cells, we performed RNA-seq analysis on purified B cells from paired PB and LN, together with B cells isolated from the PDLS generated using the same PB sample.

**Fig. 2:**
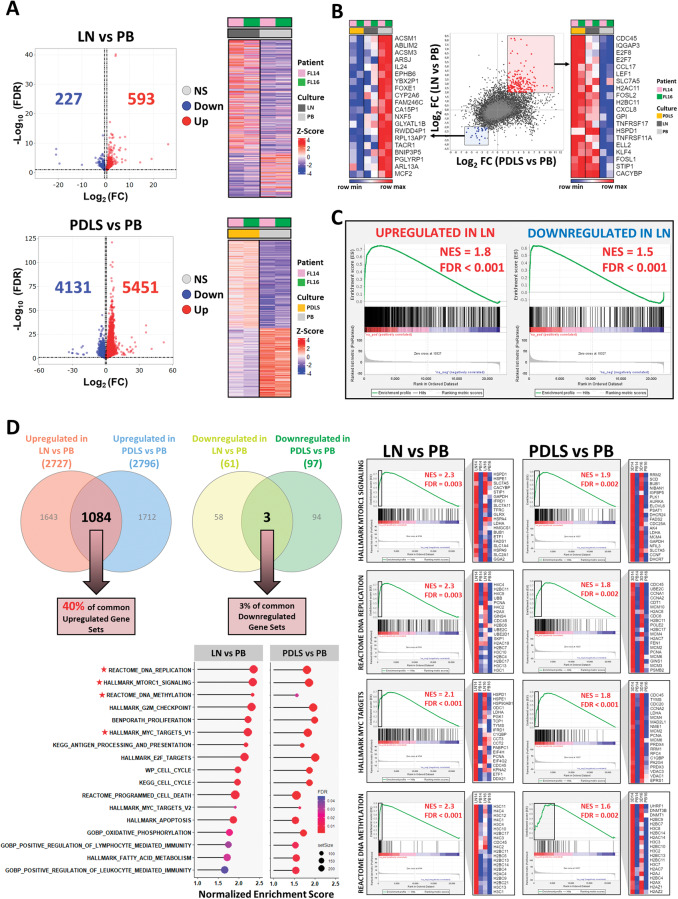
FL-PDLS transcriptome recapitulates the LN signaling. **A** Volcano plots representing the differentially expressed genes (DEG) comparing LN with the original FL peripheral blood (PB) sample (upper panel), or FL-PDLS after 7 days of culture with PB sample (lower panel). DEG was obtained by a paired (*n* = 2) DESeq2 analysis (FDR < 0.1 and absolute log_2_ Fold change (FC) > 0.5). Heatmaps of DEG for the individual patients (*n* = 2). NS non-significant, Down Downregulated, Up upregulated. **B** Scatter plot of RNA-seq comparison of LN versus PB (*y*-axis) in log_2_(fold change), and PDLS versus PB (*y*-axis), for all protein-coding genes. Heatmaps of the top-20 genes commonly upregulated (right) and downregulated (left) genes are displayed (log_2_FC > 2 or log_2_FC < −2). **C** Enrichment plot of LN signatures comparing PDLS vs PB by GSEA. **D** Venn diagrams of all significant gene set from LN vs PB and PDLS vs PB comparatives by GSEA analysis (upper left panel). Bubble plots representing the most significant and representative GSEA pathways upregulated in LN or PDLS compared to PB (lower left panel). For a selection of significantly enriched gene sets (*), GSEA plots of LN or PDLS compared to PB and a heatmap of the leading genes are represented (lower right panel).

We first performed two differential expression analyses of purified tumor B cells: (1) LN vs PB and (2) PDLS vs PB. A total of 593 genes were upregulated and 227 downregulated in LN compared to PB (Fig. 2A upper panel). This analysis allowed us the generation of LN signatures (Table S2). PDLS vs PB comparison uncovered a significant transcriptome modulation, with 5451 upregulated and 4131 downregulated genes in PDLS (Fig. 2A lower panel). We next analyzed the degree of overlap between these two comparisons as a measurement of how close PDLS were to LN. As shown in Fig. 2B, a significant number of common differentially expressed genes between LN or PDLS compared to PB were identified (199 up, 28 down) (Fig. 2B).

We next proceeded to validate if PDLS summarizes LN signaling pathways. For this purpose, we created two LN signatures including significantly upregulated and downregulated genes from the LN versus PB comparative (Fig. 2A). Using these signatures, we showed that the expression levels of the genes involved in the LN upregulated signature were also significantly upregulated in the PDLS compared to original PB (NES = 1.78, FDR < 0.001) and moderately downregulated in the case of downregulated signature (NES = 1.48, FDR < 0.001) (Fig. 2C).

To further characterize the FL-PDLS system, we conducted a GSEA analysis in the two previous comparatives (LN vs PB and PDLS vs PB), including all published gene sets in MsigDB (msigdb.v2023.1.Hs.symbols). Noteworthy, 40% of the upregulated gene sets (1084 up) in the LN vs PB comparison were also upregulated in that of PDLS vs PB. This data indicates a remarkably high homology between PDLS and LN samples (Fig. 2D upper panel). A large number of these shared gene sets were related to increased proliferation (E2F, MYC, and cell cycle-related gene sets), survival (mTOR, programmed cell death and apoptosis), metabolic pathways (OXPHOS, glucose and fatty acid metabolism) and epigenetic modifications (DNA methylation). Altogether, these findings reflect that changes that occurred during the generation of the FL-PDLS render initial PB samples to resemble an LN sample rather than the original PB (Fig. 2D lower and left panel, Table 2).

**Table 2:** Gene sets overrepresented in LN vs PB and PDLS vs PB comparatives.

Biological process	# of enriched gene sets	LN vs PB		PDLS vs PB	
		NES (max)	FDR, *q* value (min)	NES (max)	FDR, *q* value (min)
**Cellular processes**					
Apoptosis	6	2.324	0.004	1.996	0.002
Cell Signaling	4	1.860	0.029	1.864	0.002
Cellular response	11	2.407	0.004	2.049	0.002
Enzymatic activity	1	1.819	0.008	1.610	0.002
Proteasome	5	2.221	0.004	1.657	0.003
Protein transport	3	2.231	0.004	2.080	0.002
Senescence	2	2.202	0.004	1.964	0.002
Transcriptional regulation	17	2.374	0.004	1.973	0.002
**Epigenetic regulation**					
DNA methylation	7	2.339	0.004	1.992	0.002
EZH2	2	1.896	0.035	1.864	0.002
**DNA damage**					
DNA damage/repair	11	2.245	0.004	1.882	0.002
p53	3	1.995	0.004	1.654	0.003
**Immune pathways**					
Antigen presentation	1	2.168	0.004	1.688	0.002
Cytokines	35	2.345	0.004	2.056	0.002
Immune response	88	2.402	0.004	2.047	0.002
Complement	1	1.640	0.034	1.554	0.002
**Metabolic pathways**					
Fatty Acid metabolism	4	2.346	0.004	2.038	0.002
Glucose metabolism	2	1.873	0.035	1.687	0.002
Oxidative phosphorylation (OXPHOS)	5	1.772	0.015	1.724	0.002
Protein metabolism	1	1.799	0.021	1.675	0.002
RNA metabolism	1	1.945	0.013	1.679	0.002
**Proliferation**					
Cell cycle regulation	91	2.484	0.004	2.051	0.002
Cyclins	2	2.079	0.007	1.903	0.002
DNA replication	20	2.499	0.004	2.085	0.002
Kinase regulation	3	1.838	0.021	1.702	0.002
MYC regulated genes	15	2.265	0.004	1.971	0.002
RAS pathway	3	2.107	0.004	2.022	0.002
**Survival pathways**					
mTOR	3	2.341	0.004	1.857	0.002
**Others**					
Angiogenesis	1	2.093	0.004	1.753	0.002
Cytoskeleton	2	1.846	0.006	1.878	0.002
Drug response	4	2.241	0.004	2.034	0.002
Extracellular matrix	1	2.126	0.004	1.954	0.002
Hypoxia	3	2.106	0.004	1.699	0.002
Metastasis	4	2.230	0.004	1.861	0.002
Mitochondrial protein import	1	1.965	0.014	1.617	0.007
Protein folding	4	2.439	0.004	1.563	0.002
Redox balance	1	1.681	0.046	1.599	0.003
Stemness	6	1.927	0.004	1.880	0.002
Telomeres	3	2.325	0.004	1.709	0.002
Unfolded protein response	1	2.022	0.004	1.584	0.003

GSEA was used to test for significant enrichment of defined gene signatures. *NES* normalized enriched score, *FDR* false discovery rate. Threshold FDR < 0.05 and NES > 1.5. Gene sets were obtained from the MSigDB v2023.1.Hs (Mar 2023).

To explore the consequences of growing FL cells in a 3D setup, a parallel comparison was done between PDLS vs 2D culture. First, the principal component analysis (PCA) showed that samples cluster mainly by the patient (Fig. S3A). The changes were moderate and just 29 genes were upregulated and 31 downregulated in PDLS compared to standard 2D culture (Fig. S3B). These genes were related to an increase in fundamental pathways such as cell adhesion, glucose metabolism, and proliferation, while a decrease was observed in pathways related to BCR or TNFα (Fig. S3C).

Another important question was to analyze the effect of co-culturing allogeneic monocytes in comparison with a co-culture with autologous monocytes. The PCA analysis indicated that samples also cluster mainly by the patient (Fig. S4A). Focusing on gene expression analysis, we found that only 39 upregulated genes and 38 downregulated showed a differential expression (Fig. S4B) involving pathways naturally related to allograft rejection (Fig. S4C). This result demonstrates moderate variations in B cell gene expression when using allogeneic monocytes or autologous monocytes.

Altogether, these results support that FL-PDLS represents a robust 3D model recapitulating fundamental biological pathways of FL in secondary lymphoid organs such as the LN.

### FL-PDLS exhibit an immune exhaustion profile consistent with FL-LN

**Fig. 3:**
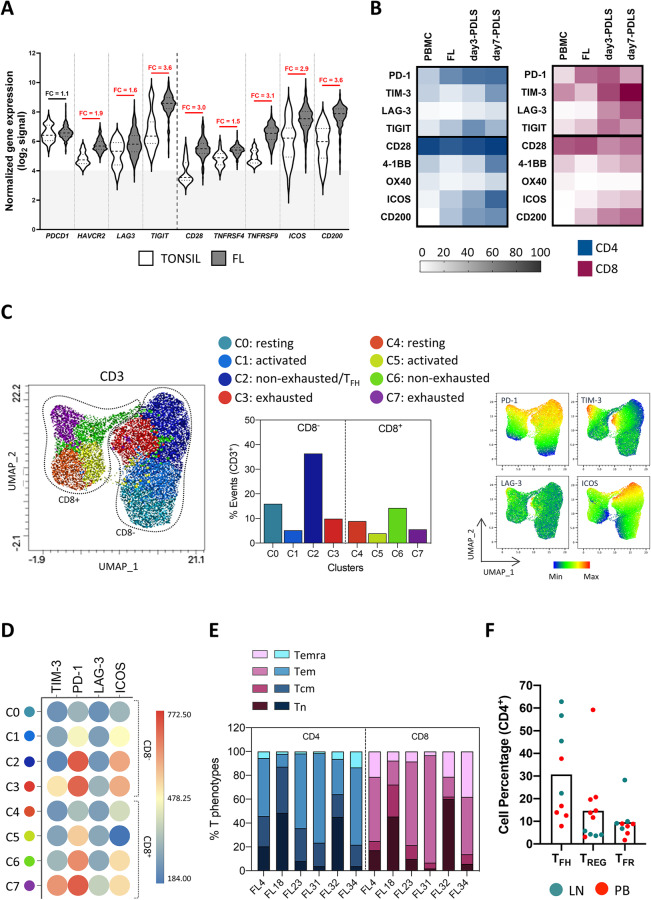
FL-PDLS immune profile. **A** Differential gene expression analysis from microarray data obtained from public repositories (detailed in supplemental methods) showed up-regulation of several immune regulators in FL-LN (*n* = 427) compared to a normal tonsil (*n* = 30). Fold changes (FC) are indicated and red color means statistical significance (unpaired nonparametric *t* test, Mann–Whitney). **B** Heatmap representing the percentage of positive cells assessed by flow cytometry for the immune regulators on CD4 and CD8 T cells of day 3-and day 7 FL-PDLS (*n* = 3–11). Results were compared with PBMCs from healthy donors (*n* = 4). **C** Uniform Manifold Approximation and Projection (UMAP) plot for day 3-FL**-**PDLS autologous CD3^+^ cells based on the expression of activation and exhaustion markers assessed by flow cytometry and colored by cluster identity (left panel). Percentage distribution of those clusters (middle panel). UMAP plots show the distribution of PD-1, TIM-3, LAG-3, and ICOS expression (right panel). **D** Average expression levels of each protein represented on CD3^+^ clusters (*n* = 6). **E** Day 3-FL-PDLS autologous CD3^+^ phenotypes based on flow cytometry CCR7 and CD45RA expression. **F** Percentage of T_FH_ (CXCR5^+^FoxP3^−^), T_REG_ (CXCR5^−^FoxP3^+^), and T_FR_ (CXCR5^+^FOXP3^+^) out of CD4^+^ population on day 3-FL-PDLS. Patients are identified by the origin of the FL sample.

Tumor-infiltrating T cells constitute the most abundant non-malignant immune population [35]. Thus, the next step was to characterize to what extent the T cells present in the FL-PDLS represent the T cell subpopulations present in FL-LN. Previous studies have described that this T cell infiltrate is composed of different levels of exhaustion governed by co-expression of specific immune checkpoints (ICs) [36–39]. We first interrogated the expression of a panel of ICs in FL biopsies and tonsils as healthy controls using available bulk RNA-seq data from public repositories. Results confirmed that ICs described in the literature encoding for the exhaustion markers *PDCD1* (PD-1), *HAVCR2* (TIM-3), *LAG-3*, and *TIGIT* were indeed increased in FL biopsies compared with the non-malignant tissue (Fig. 3A). We next validated the expression of these markers by flow cytometry at baseline (FL), and after 3 and 7 days of FL-PDLS in vitro culture. PBMCs from 4 healthy donors were used as controls (Fig. 3B). As expected, PD-1, TIM-3, LAG-3, and TIGIT were expressed in FL-PDLS at higher levels than in healthy controls and increased with culture. Interestingly, TIM-3 and LAG-3 were significantly higher in CD8 than in CD4 T cells (Fig. S4A). The differential expression analysis of FL-LN vs tonsils also highlighted IC activators such as, *TNFRSF9 (4-1BB), CD28*, and *TNFRSF4 (OX40)* (Fig. 3A). Validation of these receptors by flow cytometry showed that 4-1BB expression increased with culture, OX40 was barely expressed, and the co-stimulator CD28 was remarkably high and mostly maintained both in CD4^+^ and CD8^+^ (Fig. 3B and Fig. S5A). The costimulatory molecule ICOS, fundamental in FL-T_FH_ crosstalk, was prominent in CD4^+^ and low in CD8^+^ cells. Finally, the myeloid modulator, CD200, described to be overexpressed in FL-T_FH_ and FL [40], was highly represented in CD4^+^ and CD8^+^ cells and its expression was increased along the culture. In addition, the expression of some of their ligands was assessed in FL cells (CD20^+^) and in the myeloid compartment (CD11b^+^) within the PDLS culture (Fig. S5B). PD-L1 and CD66a (TIM-3 ligand), ICOSL and OX40L were represented in both populations, while 4-1BBL was barely detected neither in CD20^+^ nor CD11b^+^.

To better characterize the immune exhaustion of the T cell compartment in the PDLS, we analyzed the co-expression of PD-1, TIM-3, LAG-3, and ICOS on CD4^+^ and CD8^+^ T cells (day 3-PDLS) using bioinformatics tools to cluster different CD3^+^ phenotypes based on flow cytometry expression (Fig. 3C, D). Eight clusters were identified, divided by a clear polarized expression of the CD8 marker. Within each population (CD3^+^CD8^-^ and CD3^+^CD8^+^), four clusters were identified (C0–C3 and C4–C7, respectively) representing the T cell phenotype spectrum from non-activated or resting populations to terminally differentiated or exhausted (Fig. 3C, D).

Moreover, using CCR7 and CD45RA markers (Fig. 3E), we could confirm that our system recapitulates the whole spectrum of the T cell phenotypic states and the heterogeneity seen in FL scRNA-seq studies [35]. On average and consistent with other NHL studies [41], T effector memory (Tem) represented the main subset both in CD4 and CD8 T cells (mean: 48.57 and 49.56, respectively), T naïve (Tn) were also equally represented between CD4 and CD8 (mean: 19.84 and 21.84, respectively). Interestingly T central memory (Tcm) cells were more abundant in the CD4 population, while Terminally differentiated T cells (Temra) were in CD8 T cells (Fig. S6A).

Finally, considering the remarkable role of CD4^+^ cells in the FL-TME [13, 18], the presence of T_FH_, and the regulatory subsets, T_REG_ and T_FR_, were confirmed in all PDLS analyzed (Fig. 3F and Fig. S6B), albeit with different abundance depending on the patient material. LN-derived PDLS were significantly enriched in T_FH_, while PB-derived PDLS were, in most cases, enriched with T_REG_.

In this regard we characterized the evolution of these subpopulations within FL-PDLS (day 3), compared with the unstimulated sample (day 0), together with their ICs. We performed this analysis separately in FL-PDLS generated from PB and LN samples (Fig. S6C). Compared with the corresponding unstimulated samples, T_REG_ increased, and T_FH_ decreased in PB-PDLS, while T_FH_ and T_FR_ increased in LN-PDLS. T_FH_ clearly gains prominence in LN-PDLS, maintaining the expression of PD1 and increasing LAG3 and TIM3, while this observation does not apply to PB-PDLS. T_FR_ constitutes the population with a more immune suppressive profile in both PB and LN-derived PDLS, suggesting a common evolution with the culture conditions, albeit of the origin of the sample.

Altogether we have demonstrated that the T cell compartment in FL-PDLS recapitulates features of the FL–LN microenvironment with different T cell subsets encompassing a spectrum of activation/exhaustion phenotypes and preserving the main T cell subpopulations found in FL–LN.

### PDLS represents a suitable system for immunotherapy screening

**Fig. 4:**
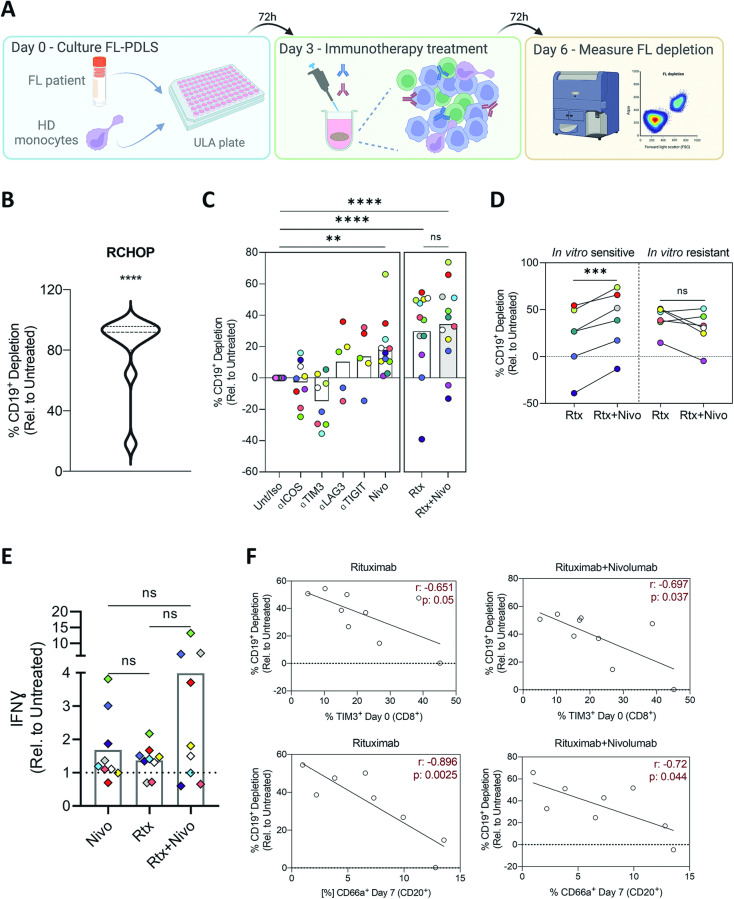
FL-PDLS represents a suitable system for immunotherapy drug screening. **A** Schematic representation of the workflow used to treat FL-PDLS with immunotherapeutic agents. HD healthy donors. Created with BioRender.com. **B** Percentage of B cell depletion induced by R-CHOP or **C** a sort of selected monoclonal antibodies (IgG1κ mAbs) against IC of interest, and nivolumab (Nivo) combined with rituximab (Rtx) relative to the untreated/Isotype condition (Unt/Iso). Depletion was assessed by Aqua^+^ cell counting. **D** Percentage of B cell depletion for rituximab (Rtx) and its combination with nivolumab (Rtx+Nivo) of in vitro responder (R) and non-responder (NR) patients. Patients were considered responders when reaching a Response Index* superior to the median value of 18%. Patient coding is included in Table 1. **E** IFNɣ levels (pg/mL) were measured in the day 6-PDLS supernatants relative to the untreated condition (right panel). Quantification was done using the CBA application. Each supernatant was recovered from 6 replicates per condition and patient. Patient coding is indicated. **F** Correlation plots by simple linear regression of B cell depletion in FL-PDLS treated with rituximab or rituximab + nivolumab and the expression of TIM-3 in CD8+ cells at basal levels (day 0) and CD66a in B cells at day 7 of culture. Paired *t* test–Wilcoxon matched-pairs signed rank test was applied in (**B**) and (**D**), and one-way ANOVA followed by Holm–Sidak post hoc test for (**C**) and (**E**). ${{\ast}\atop } {{\rm{Response}}\; {\rm{index}}}\,{{=}}\,\left(\frac{{\rm{B}}\,{\rm{cell}}\,{\rm{depletion}}\,\left({\rm{Rtx}}\,{{+}}\,{\rm{Nivo}}\right)\,{{-}}\,{\rm{B}}\,{\rm{cell}}\,{\rm{depletion}}\,({\rm{Rtx}}\,)}{{\rm{B}}\,{\rm{cell}}\,{\rm{depletion}}\,({\rm{Rtx}})}\right){{* }}{{100}}$.

We next sought to demonstrate that FL-PDLS may be a valuable tool for personalized medicine. In the established workflow (Fig. 4A), treatments were applied to day 3-PDLS, when the spheroid has compacted, and proliferation has been engaged. Treatment efficacy was assessed after 3 days (day 6) by measuring B cell depletion. We first tested the FL first-line treatment R-CHOP, and we observed an induction of tumor cell depletion above 80% in 9 out of 11 FL-PDLS (81%) (Fig. 4B, Figure S7A) that is consistent with the observed response rate in the clinical practice [42].

We next challenged FL-PDLS to several immunotherapeutic agents targeting ICs described before, together with the standard therapy rituximab. Antibodies targeting ICOS, TIM-3, LAG-3, or TIGIT engaged mostly limited and highly heterogeneous responses (Fig. 4C). Anti-PD-1 (nivolumab/Nivo) was the most effective IC inhibitor that also increased rituximab activity in half of the FL-PDLS (6/12) (Fig. 4D). This combination increased the levels of IFNɣ in the PDLS supernatants compared to single agents in five out of the nine cases analyzed, albeit without reaching statistical significance (Fig. 4E).

These heterogeneous responses to IC inhibitors are consistent with observations in the clinic and may be explained by the patient-specific composition and activation/exhaustion status of the T cell compartment [43, 44]. In this regard, correlation analyses indicated that depletion induced by rituximab or rituximab combined with nivolumab inversely correlated with the expression of TIM-3 on CD8 T cells and the expression of TIM-3 ligand, CD66a, on B cells (Fig. 4F). It has also been demonstrated that TIM-3 is highly expressed by T_REG_ and promotes T cell dysfunction in several cancers [45]. In our system, we observed that those PDLS with a higher percentage of T_REG_ prior in vitro rituximab treatment responded worse than those with lower proportions (Fig. S7A). In addition, the percentage of T_REG_ in the PDLS correlated with the expression of TIM-3 in CD4 T cells (Fig. S7B), suggesting a possible relation between TIM-3^+^ T_REG_ and therapy resistance.

In summary, FL-PDLS represents a valuable multiplexed patient-derived system with great potential for personalized immunotherapeutic approaches and for the identification of biomarkers of response and resistance.

### Inhibition of galectin-9 but not TIM-3 improves rituximab FL depletion

**Fig. 5:**
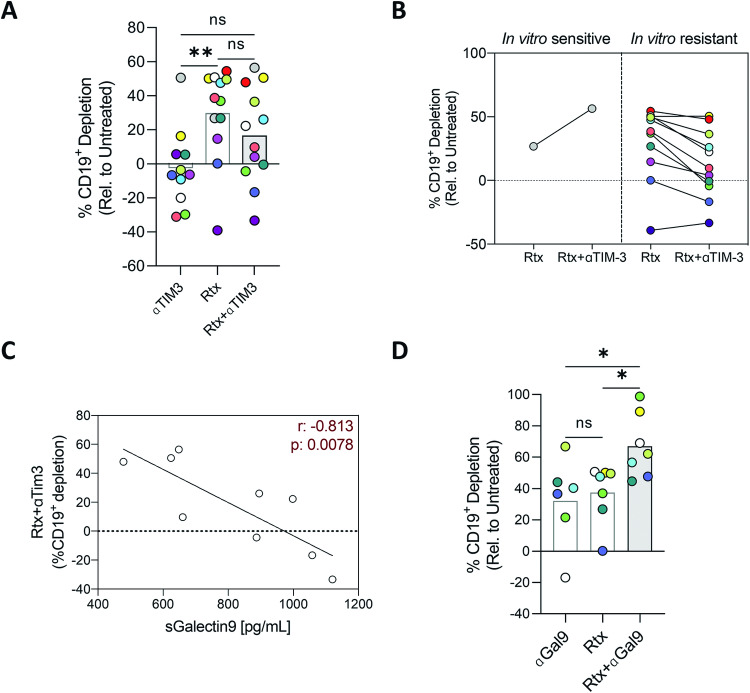
Galectin-9 blockade improves rituximab-induced depletion in FL-PDLS. **A** B cell depletion induced by mAb anti-TIM-3 (αTIM-3), rituximab (Rtx), or the combination (Rtx + αTIM-3) in all patients and, **B** classified in in vitro responder (R) and non-responder (NR) patients to the combination. Patient coding is included in Table 1. **C** Correlation by simple linear regression between galectin-9 levels (pg/mL) analyzed by ELISA of FL PDLS supernatants at the endpoint (day 6) and the B cell depletion induced by rituximab + anti-TIM-3. **D** B cell depletion induced by mAb anti-galectin-9 (ɑGal9), rituximab (Rtx), or the combination. One-way ANOVA followed by Holm-Sidak post hoc test was applied for (**A**) and (**D**).

Based on the possible role of TIM-3 on rituximab activity, it was conceivable that TIM-3 blockade may improve those limited responses. However, combination therapy did not offer any benefit in terms of FL depletion (Fig. 5A). On the contrary, a reduction of rituximab activity was observed in most of the cases analyzed (Fig. 5B). This may be explained by the fact that TIM-3 has multiple ligands: CEACAM1 (CD66a), Phosphatidylserine (PtdSer), High Mobility Group Protein B1 (HMGB1) and galectin-9, which bind to different regions of the receptor [46]. More importantly, it was discovered that antibodies targeting TIM-3 bind to the FG–CC’ loops of its extracellular domain but are incapable of blocking the glycan binding site where galectin-9 binds [47] (Fig. S8A). Thus, considering the correlation between therapy resistance and T_REG_ percentages in our cultures, the inactivity of anti-TIM-3 antibodies against galectin-9 interactions, and the growing evidence of galectin-9 role in tumor immune escape [48, 49], we moved to explore galectin-9 as an interesting target to improve rituximab-induced depletion. We first analyzed soluble galectin-9 (sGalectin-9) in FL-PDLS supernatants, and we observed a negative correlation with rituximab + anti-TIM-3 FL depletion (Fig. 5C). Moreover, high levels of sGalectin-9 also have a negative impact on rituximab and rituximab + nivolumab induced depletion (Fig. S8B). We next demonstrated that anti-galectin-9 mAb was able to induce FL depletion as a single agent and significantly improved rituximab activity (Fig. 5D), frequently decreasing TIM-3 expression in CD4^+^ and CD8^+^ populations (Fig. S8C). Nevertheless, FL depletion negatively correlates with the presence of T_FH_ (Fig. S8D).

Altogether, using our novel FL-PDLS tool, we have uncovered galectin-9 as a new IC with potential interest for FL immunotherapy that may deserve attention to improve standard anti-CD20 immunotherapy or in combination with other IC modulators.

## Discussion

Robust patient-derived models constitute one of the fundamental pieces for the successful implementation of personalized medicine. We have previously established 2D cocultures with FDC [50, 51] and Mϕ [23] and demonstrated their utility in studying FL-microenvironment crosstalk, therapy testing, and biomarker identification. Yet, 3D models better represent cancer biology, signaling pathways [52], and especially B and T cell activation, as they are influenced by physical forces that are not recapitulated in 2D cultures [53]. We have developed FL 3D models using cell lines alone or in co-culture with immune cells [54–58]. In addition, we have recently made progress in patient-derived FL 3D models, including both tumor cells and the T cell compartment [27]. However, this model does not include myeloid cells also fundamental for lymphoma maintenance and immune suppressive signaling. For these reasons, we endeavored to generate the first immunocompetent patient-derived FL 3D model, including B, T, and myeloid compartments. Future perspectives we are approaching to improve and complexify this system include the integration of FDC cells and ECM, together with the inclusion of these tumoroids in a microvascularized system.

The FL-PDLS model we present here is an affordable and robust system for the following reasons:

First, using PBMCs from PB or LN samples of FL patients, we optimized a cytokine cocktail to mimic the underlying interactions between FL-T_FH_ (CD40/CD40L, IL-4, and IL-21) [17–19]. The addition of IL-15 together with the monocytes completes the main immunological actors. IL-15 activates both NK and T cells, while the presence of monocytes/macrophages, together with CD40/CD40L engagement, can activate B cells [31]. Due to the low number of monocytes in the original FL-PB sample and as macrophages are not recovered with the LN mechanical dissociation performed in the clinical routine, we decided to introduce monocytes from healthy donors in a conservative ratio since they represent an elemental compartment that constitutes a source of immunosuppressive signal [23]. Because of the proliferation engaged by B and T cells in the FL-PDLS, the monocyte ratio decreases after several days (mean population distribution in day 7-FL-PDLS: 60.11% CD20^+^, 35.27% CD3^+^, and 4.62% CD11b^+^). It is noteworthy that in the FL-PDLS system, monocytes started a process of differentiation and polarization in the absence of specific differentiation factors, with some limitations due to the absence of ECM that enables their adhesion. We have previously reported that FL cells secrete M-CSF that facilitates Mn differentiation. In the FL-PDLS, the presence of IL-4 (in the PDLS medium) and IL-10 generated by FL-Mϕ crosstalk [23] may contribute to the acquisition of a phenotype with some markers of immunosuppressive M2-Mϕ, as seen in FL-LN biopsies [22], while CD40L signaling would enhance the expression of M1 markers.

Second, we have demonstrated that B cells in the FL-PDLS recapitulate fundamental transcriptional pathways in FL-LN. To do so, we have performed a 3-way transcriptomic comparison of B cells isolated from paired LN, PB, and PB-derived FL-PDLS. With this approach we have demonstrated that B cells in FL-PDLS are committed to a LN transcriptomic program through the enrichment of LN signatures and the activation of pathways overrepresented in LN compared to paired PB. These pathways include proliferation, mTOR pathway, epigenetic regulation, diverse metabolic pathways and housekeeping processes, overall reflecting that FL-PDLS is a living and dynamic system. Moreover, RNA-seq analysis highlighted the activation of immune pathways in the FL-PDLS as seen in the LN (antigen presentation, IFNγ, TGFβ, IL-2, and IL-10 signaling). Altogether, our results are in agreement with previous scRNA-seq studies showing common gene set enrichments in B cells from FL-LN compared to normal B cells [59].

Third, FL-PDLS recapitulates the immune exhaustion profile of FL-LN. We have demonstrated the previously acknowledged high expression of immune regulators such as TIM-3, LAG-3, TIGIT, ICOS, or CD200. More importantly, the analysis of the IC co-expression, together with the activation phenotype (Tn, Tem, Tcm, Temra), allowed us to confirm that FL-PDLS fairly recapitulates the spectrum of CD8 clusters recently acknowledged in FL studies and the interpatient variability [35]. This feature is mandatory to use FL-PDLS as a system for immunotherapies testing.

Thus, we decided to challenge FL-PDLS with rituximab + anti-PD-1 combination as this regimen has demonstrated favorable efficacy (ORR; 67%; CR:50%) and safety profile in relapsed/refractory FL [60]. In the FL-PDLS system, 50% of the cases benefit from the combination, and resistance was related to CD8 exhaustion, a feature also acknowledged in the correlative analysis of the above-mentioned clinical trial. In our FL-PDLS system, we observed that the TIM-3 axis may have a role in this CD8 exhaustion since its expression in CD8 and the expression of its ligand CD66a in B cells correlated with reduced responses to rituximab or rituximab + nivolumab combination. However, when we challenged FL-PDLS with rituximab + mAb anti-TIM-3 combination, we obtained unforeseen results with a general decrease in antitumoral activity. TIM-3 has multiple ligands, among them, galectin-9 is gaining relevance in cancer [61]. Galectins are a family of endogenous glycan-binding proteins that reprogram tumor, vascular, and immune landscapes in the tumor microenvironment [62, 63]. Moreover, galectins dampen antitumor immune responses by targeting both lymphoid and myeloid cells. In the precise case of galectin-9, expression can be up or downregulated in association with neoplastic transformation depending on the specific tumor type [61]. Galectin-9 is increased in several hematologic neoplasia, including chronic lymphocytic leukemia [64], cutaneous T cell lymphoma, and acute myeloid leukemia (AML) [65]. In this latter, TIM-3/Gal-9 constitutes an autocrine stimulatory loop that regulates the self-renewal of leukemic cells [66]. Importantly, anti-TIM-3 mAbs are incapable of blocking the binding site where galectin-9 binds, and a specific mAb is required. In this regard, two clinical trials with the mAb anti-Gal9 (LYT-200) are currently ongoing for AML (NCT05829226) and advanced solid tumors (NCT04666688). In FL-PDLS, we have demonstrated that sGalectin-9 is related to reduced responses to rituximab or rituximab + nivolumab, and to the lack of activity observed in rituximab + mAb anti-TIM-3 combination. Finally, mAb blocking galectin-9 offered a therapeutic benefit in combination with rituximab in all cases analyzed. Additionally, those patients with limited responses to rituximab + anti-galectin-9 combination showed a higher proportion of T_FH_, known to overexpress PD-1. These observations suggest that co-blockade of galectin-9 and PD-1 could benefit rituximab combinations in those patients with high numbers of T_FH_.

In summary, we have set up a robust patient-derived system, recapitulating LN signaling in FL cells, the spectrum of T cell phenotypes, and the presence of main T cell subpopulations. We have demonstrated that FL-PDLS represents a novel tool for the discovery of immunotherapeutic targets, such as galectin-9. FL-PDLS may serve as a platform to perform preclinical screening in a robust, 96-well format system and may also constitute a complementary in vitro tool for phase 1/2 trials to help identify biomarkers of response and mechanisms of resistance.

## Supplementary information

Supp Video 1

Supp Video 2

supp material

Table S1

table S3

Table S2

## Data Availability

RNA sequencing data generated and analyzed during the current study are available in the European Genome-phenome Archive (http://ebi.ac.uk/ega/) under accession number EGAS50000000233. The datasets generated and/or analyzed during the current study are available from the corresponding authors upon reasonable request.
