# Supplementary material for: Patient-derived follicular lymphoma spheroids recapitulate lymph node signaling and immune profile uncovering galectin-9 as a novel immunotherapeutic target
Source: Blood Cancer J. 2024 May 2;14(1):75. doi: 10.1038/s41408-024-01041-7 (PMC11636880; doi:10.1038/s41408-024-01041-7)
Supplement: Supplementary file 3 — supp material [file 41408_2024_1041_MOESM3_ESM.pdf]

SUPPLEMENTAL METHODS

Patient samples and healthy donor peripheral blood monocytes isolation

FL primary samples, diagnosed according to the World Health Organization classification criteria in the Hematopathology Unit of Hospital Clinic of Barcelona (HCB, Barcelona, Spain), were isolated from peripheral blood (PB) and tumor lymph nodes (LN) biopsies. These samples belong to Hematopathology collection, registered in the Biobank of IDIBAPS-Hospital Clinic, Barcelona (R121004-094) and in the National Registry of Biobanks-ISCIII (C.0000397). Patients had signed an informed consent approved by the Ethics Committee of HCB and the Declaration of Helsinki. Patient clinical features are summarized in Table 1. Monocytes from healthy donors were isolated from peripheral blood mononuclear cells (PBMCs) of buffy coats provided by Banc de Sang i Teixits (Barcelona, Spain). After Ficoll-Paque gradient separation (GE Health Care, Chicago, IL, USA), monocytes were purified using $CD14^+$ human magnetic beads and LS columns (Miltenyi Biotec, Bergisch Gladbach, Germany). Purity >96% was verified by flow cytometry (FACS Fortessa (BD Biosciences)) before cryopreservation with 10% DMSO (Sigma-Aldrich, St. Louis, MO, USA).

FL-PDLS immune profile

The immune profile of FL samples after thawing (day 0), at day 3 (day 3-PDLS) and day 7 (day 7-PDLS) was characterized using flow cytometry panels for B ($CD20^+$) cells, T ($CD4^+$ and $CD8^+$) cells and monocytes/macrophages ($CD11b^+$) including the following antibodies against: TIM-3, PD-1, TIGIT, ICOS, LAG-3, CD28, 41BB, OX40, CD200, ICOSL, CD66a, PD-L1, OX40L, 41BBL, CD200R. PBMCs (n=4) from healthy donors were used as controls. T cell phenotype was characterized by the expression of CCR7 and CD45RA. FoxP3 intracellular staining together with CXCR5 were used to characterize T_{FH}, T_{REG} and T follicular regulatory cells (T_{FR}) subsets. The study of $CD3^+$ clusters (C0-C7) based on the expression of CD8, PD-1, TIM-3, LAG-3 and ICOS was assessed using FlowJo (BD Biosciences, Franklin Lanes, NJ, USA) plugins using data from 6 patients. Cluster Explorer after dimensionality reduction (UMAP) (1) and hierarchical clustering (FlowSOM) (2) creates an interactive cluster profile graph and heatmaps.

Additionally, frozen supernatants from day 6-PDLS samples were used to quantify soluble galectin-9 concentration by ELISA (Thermo Fisher) following the manufacturer's instructions. Data was collected using the colorimetric microplate reader Infinite® 200 PRO (Tecan, Männedorf, Switzerland).

FL-PDLS imaging

PDLS formation has been tracked by brightfield illumination on the automated digital microscope Cytation 1 Cell Imaging Multi-Mode Readers (BioTek, Agilent, Santa Clara, CA, USA) and analyzed with Gen5 software (Biotek). Captures were performed under 37ºC temperature and 5% CO_2 using Z-stacking function (n=3).

3D captures and analysis were performed with SPIM (ZEISS Lightsheet Z.7, Imactiv 3D) and image-processing algorithms were developed in MATLAB. Day 7-PDLS were fixed in 4% paraformaldehyde (PFA (Electron Microscopy Sciences, Hatfield, PA, USA)) overnight (ON) at 4ºC. Then, after three rinses, PFA was diluted to 0.5% with PBS and labeled with 10 µg/mL Propidium Iodide (Thermo Fisher Scientific) for 4h at room temperature (RT) with agitation. After the incubation, PDLS were washed three times with PBS, included in 1.5% agarose and cleared using methanol and Benzyl Alcohol/Benzyl Benzoate (BABB) reagent.

Immunofluorescence

PDLS populations had been captured using confocal microscopy (LEICA TCS SPE, Wetzlar, Germany) and analyzed with ImageJ software (NIH, Bethesda, MD, USA). After fixation and several rinse cycles, PBS was finally replaced by permeabilization/blocking (P/B) buffer (PBS + 2% FBS + 2% BSA + 0.6% Triton + 0.01% Azide) and incubated ON with agitation at RT. PDLS were then labeled with 1:100 CD19 (Invitrogen), 1:100 CD3 (Bioss Antibodies, Wobrn, MA, USA) and 1:100 Ki-67 (Dako Omnis, Santa Clara, CA, USA) anti-human antibodies under agitation at RT ON. Monocytes were stained before being cultured, with Far Red CellTrace (Thermo Fisher Scientific) to be localized in the spheroid. After 3 washes with P/B buffer, PDLS were labeled with a mixture of 1:500 anti-Rat + 1:500 anti-Rabbit + 1:500 anti-Mouse secondary antibodies (Thermo Fisher Scientific) ON at RT under agitation. After the incubation, PDLS were washed three times with PBS, included in 1.5% agarose and cleared using methanol and Benzyl Alcohol/Benzyl Benzoate (BABB) reagent.

BCL2 rearrangement was assessed by Fluorescence in situ hybridization (FISH) using *BCL2* break-apart and/or *IGH*/*BCL2* dual-color dual-fusion commercial probes (Metasystems, Altlussheim, Germany). The hybridization was performed according to the manufacturer's protocols. At least 100 nuclei were examined for each probe whenever possible. Digital image acquisition, processing, and evaluation were performed using ISIS digital image analysis version 5.0 (MetaSystems Barcelona, Spain).

Monocyte-Macrophage differentiation and polarization analysis

To assess differentiation and polarization of the myeloid compartment included in the day 7-PDLS, $CD11b^+$ Far Red^+ cells were purified (over 96.0%) and recovered by cell sorting (FACS Aria, BD Biosciences (Cytometry and cell sorting facility, IDIBAPS, Barcelona, Spain)) and RNA was isolated with TRIZol following manufacturer's protocol (Thermo Fisher Scientific). cDNA was synthetized using Preamp RT Master Mix (Fluidigm, San Francisco, CA, USA). Next, cDNA was pre-amplified for the following genes: *CXCL11, CCL5, MRC1, CCL22, PMAIP1, RSG2, GUSB,* using pre-designed TaqMan probes (Thermo Fisher Scientific) and PreAmp Master Mix (Fluidigm).

Quantitative real-time PCR (qPCR) was then performed on a StepOne Real-Time PCR System (Thermo Fisher Scientific) using the same probes as the preamplification. Samples were analyzed in duplicate, and expression was normalized using the housekeeping gene *GUSB.* To evaluate the degree of M1/M2 polarization FL-macrophages were compared with M1 or M2-polarized macrophages generated as follows: monocytes obtained from PBMCs as previously described were seeded in 6-wells plates at $0.5x10^6$ cells/mL in enriched medium (EM) supplemented with macrophage colony stimulating factor (M-CSF) (Thermo Fisher Scientific) at 100 ng/mL to differentiate them to macrophages (M0). After 6 days, additional stimuli were provided to obtain M1-polarized macrophages (20 ng/mL IFNγ (Gibco, Thermo Fisher Scientific) + 100 ng/mL LPS (Sigma-Aldrich)) or M2-polarized macrophages (20 ng/mL IL-4 (PeproTech, Rocky Hill, NJ)) for 24h.

RNA-seq and data analysis

Day 7-PB-PDLS were disaggregated, labeled with LIVE/DEAD Aqua, CD20 (Thermo Fisher Scientific) and CD3 (BD Biosciences). FL-B cells ($CD20^+$ $CD3^-$ FR^- $Aqua^-$) were recovered using a BD FACSARIA II sorter (Cytometry and cell sorting facility, IDIBAPS) and RNA extracted as previously mentioned. Likewise, FL-B cells ($CD20^+$ $CD3^-$ FR^- $Aqua^-$) isolated from the original paired FL-PB and FL-LN sample from the same patient at thawing (day 0) were used as a comparator for RNA-seq studies. Purity was over 95%.

RNA was assayed for quantity and quality using Qubit RNA HS Assay (Thermo Fisher Scientific) and RNA 6000 Nano Assay on a Bioanalyzer 2100 (Agilent). Stranded RNA-seq libraries were performed for 150 ng of mRNA using the TruSeq library kit (Illumina, San Diego, CA, USA). Libraries were sequenced on a NextSeq2000 (Illumina) in a 2x50bp length with a coverage of >40 million paired-reads per sample.

Sequencing reads were trimmed using trimmomatic (version 0.38) (3) and ribosomic RNA reads were filtered out using SortMeRNA (version 2.1b) (4). Gene-level counts (GRCh38.p13; Ensembl release 100) were calculated using kallisto (version 0.46.1) (5) and tximport (version 1.6.0) (6). Paired differential expression analyses were conducted using DESeq2 (version 1.18.1) (7). All samples were used to estimate size factors and dispersions. Shrinkage of effect size was performed using the ashr method (8). Adjusted *P* value (*Q*) <0.10 and absolute log2-transformed fold change >0.5 were used to identify differentially expressed genes (DEGs).

Gene-set enrichment analyses (GSEAs) were conducted with GSEA software (version 4.3.2) (9) using the pre-ranked modality and log2FC results obtained from DESeq2 as input data. All Human collections of MSigDB gene sets were used (Human MSigDB v2023.1.Hs updated March 2023). Heatmaps of selected genes were generated using R and Morpheus software.

Metadata comparative analysis

mRNA relative expression levels of selected immune checkpoints in whole lysates of follicular lymphoma lymph nodes (FL-LN, n=427) was compared with normal tonsils (TONSIL, n=30) according to GEP public databases (GSE3526, GSE7307, GSE31311, GSE39503, GSE43346, GSE65136, GSE71810, GSE53820, GSE55267, GSE86622, GSE93261, GSE12453, GSE12195, GSE35426 and GSE132929). This selected data was all generated with Affymetrix Human Genome U133 Plus 2.0. Briefly, CEL files were normalized using the Expression Console TM Software v1.4.1.46 (Affymetrix, Santa Clara, CA, USA). To take in consideration the batch effect, joint data was normalized using the Limma package included in Transcriptome Analysis console (Applied Biosystems, Waltham, MA, USA).

Statistical analysis

Data were analyzed using Prism Software 9.0 (GraphPad Software, San Diego, CA, USA). We used non-parametric test t-test, paired (Wilcoxon test) or unpaired (Mann-Whitney test) depending on the experiment. Likewise, ordinary one-way ANOVA followed by Holm-Sidak post hoc test was used for multiple comparisons. Data were represented as the mean values of the patients analyzed and standard deviation. Pearson correlation coefficient was used to measure linear correlations. Differences between the results of comparative tests were considered significant if the two-sided P value was less than 0.05. The statistical significance convention used along the manuscript was as follows: * $p<0.05$, ** $p<0.01$, *** $p<0.001$ and **** $p<0.0001$.

BIBLIOGRAPHY

1. McInnes L, Healy J, Melville J. UMAP: Uniform Manifold Approximation and Projection for Dimension Reduction. Arxiv - Cornell Univ [Internet]. 2018 Feb 9; Available from: http://arxiv.org/abs/1802.03426
2. Gassen S Van, Callebaut B, Helden MJ Van, Lambrecht BN, Demeester P, Dhaene T, et al. FlowSOM : Using Self-Organizing Maps for Visualization and Interpretation of Cytometry Data. Cytometry. 2015;87(7):636–45.
3. Bolger AM, Lohse M, Usadel B. Trimmomatic: a flexible trimmer for Illumina sequence data. Bioinformatics. 2014;30(15):2114–20.
4. Kopylova E, Noé L, Touzet H. SortMeRNA: fast and accurate filtering of ribosomal RNAs in metatranscriptomic data. Bioinformatics. 2012;28(24):3211–7.
5. Bray NL, Pimentel H, Melsted P, Pachter L. Near-optimal probabilistic RNA-seq quantification. Nat Biotechnol. 2016;34(5):525–7.
6. Soneson C, Love MI, Robinson MD. Differential analyses for RNA-seq: transcript-level estimates improve gene-level inferences [version 2; peer review: 2 approved]. F1000Research. 2016;4(1521).
7. Love MI, Huber W, Anders S. Moderated estimation of fold change and dispersion for RNA-seq data with DESeq2. Genome Biol. 2014;15(12):550.
8. Sthephens M. False Discovery rates: a new deal. Biostatistics. 2017 Apr 1;18(2):275-294.
9. Subramanian A, Kuehn H, Gould J, Tamayo P, Mesirov JP. GSEA-P: a desktop application for Gene Set Enrichment Analysis. Bioinformatics. 2007;23(23):3251–3.

SUPPLEMENTAL FIGURES

Figure S1

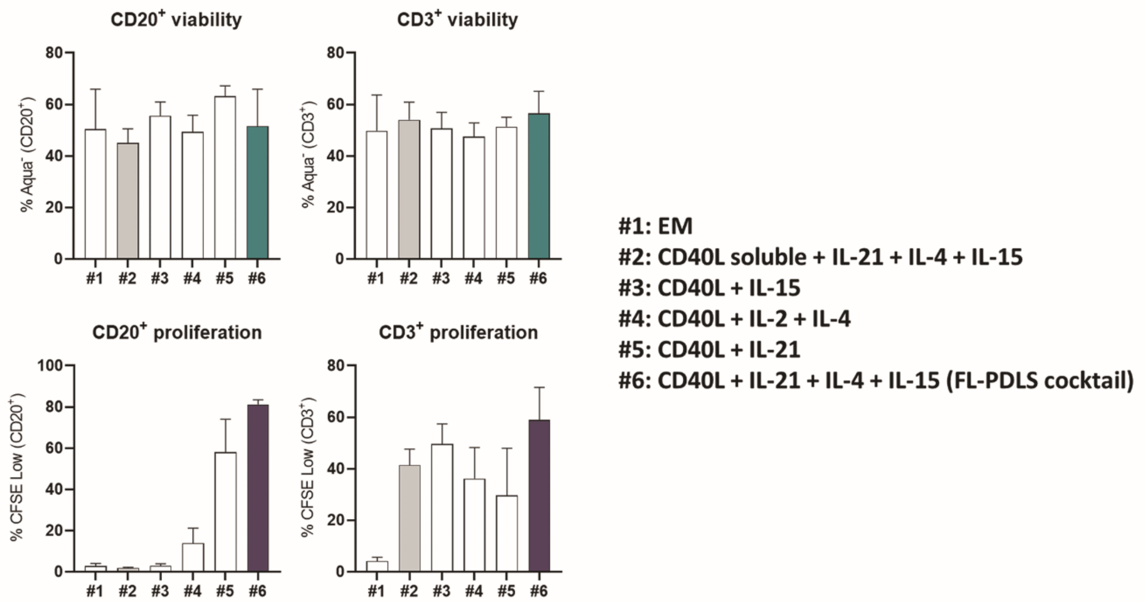

Figure S1. Optimization of PDLS cocktail. PDLS (n=10) including normal donor monocytes were cultured with the indicated cytokine cocktails. $CD20^+$ and $CD3^+$ population viability (upper panel) was determined by the percentage of $Aqua^-$ flow cytometry staining, and proliferation (lower panel) was analyzed by the percentage of CFSE low signal after 7 days of culture.

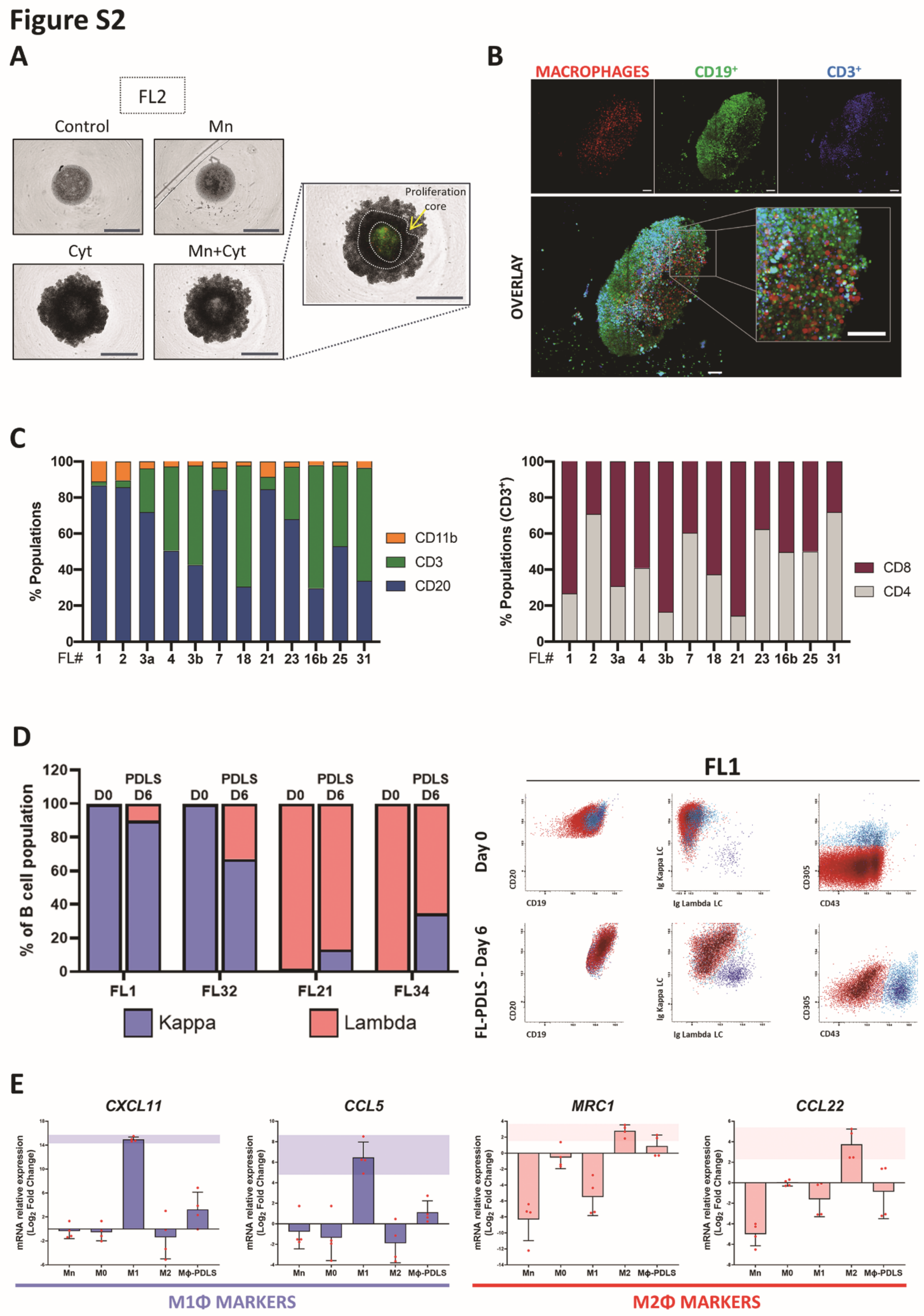

Figure S2. FL-PDLS additional features. (A) Brightfield images superimposed with CFSE (Cytation 1) in FL-PLDS after 7 days of culture with the following conditions: non-stimulated (Control), cytokine cocktail (Cyt), with monocytes (Mn) and the complete condition combining both cytokines and monocyte co-culture (Mn+Cyt). Magnification 4x and 1000 µm scale. **(B)** Immunofluorescent capture of CD19$^+$ (green), CD3$^+$ (blue) or Far Red (macrophages(red)) labeled cells, and merged image of all 3 channels (overlay). Captured in confocal Leica TCS SPE microscope. Scale 100 µm. **(C)** CD20$^+$, CD3$^+$ and CD11b$^+$ cell proportions (left panel) and CD4$^+$, CD8$^+$ out of CD3$^+$ (right panel) from day 7-FL-PDLS. **(D)** Light chain restriction analysis in four representative FL-PDLS. Kappa/lambda percentages are shown in the control sample (D0) and in the corresponding FL-PDLS (day 6). Representative density plots indicating the population (CD19+ CD20+), the light chain distribution and the expression of CD305 and CD43 in D0 sample and in the corresponding FL-PDLS. **(E)** Gene expression of M1 and M2 makers by RT-qPCR in CD11b$^+$ cells sorted from day 7-FL-PDLS, compared to Mn, M0, M1 or M2 macrophages. Values are relative to M0 macrophages (mean, n=4).

Figure S3

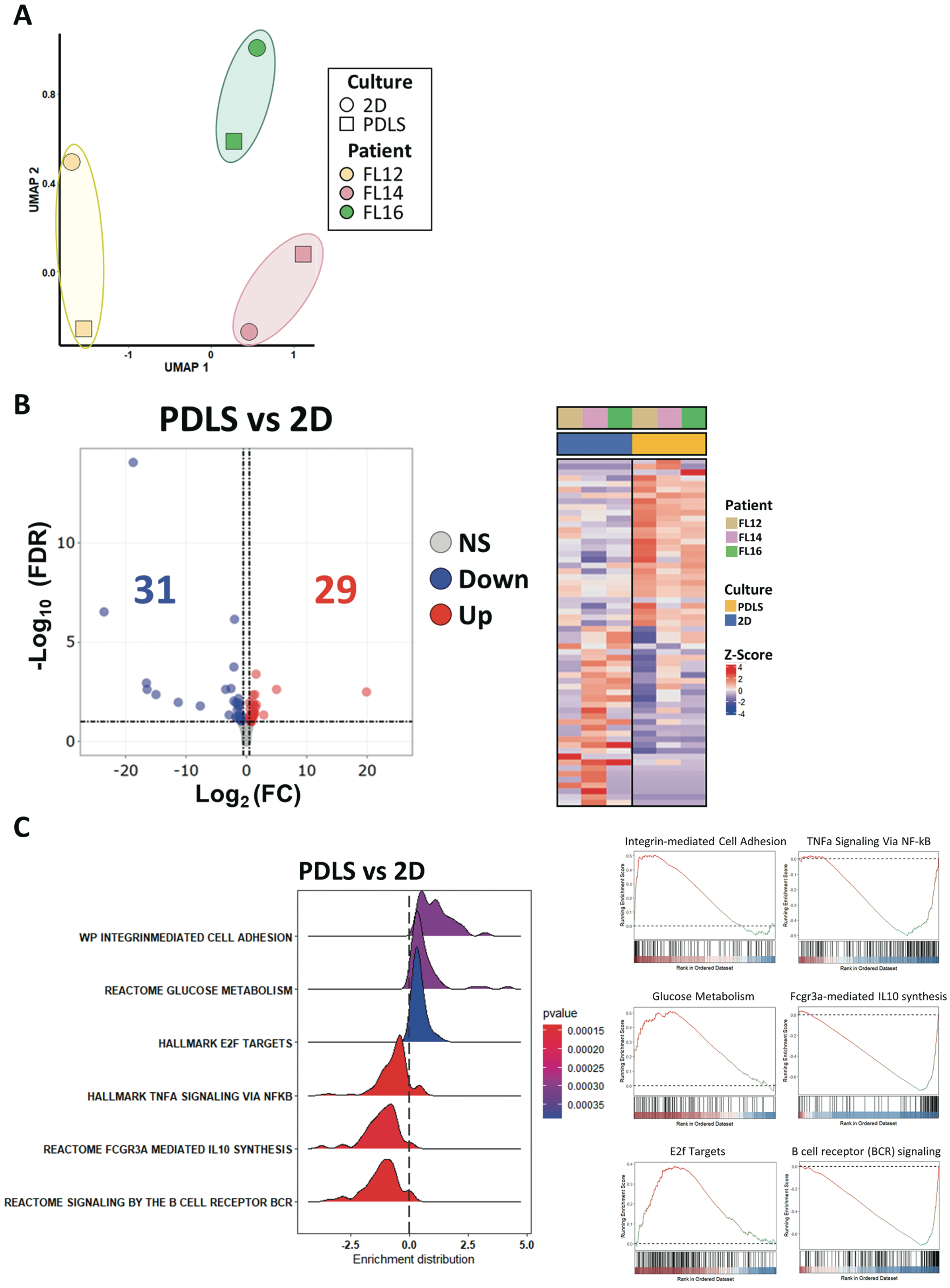

Figure S3. FL-PDLS and 2D comparison. (A) Purified B cells from FL-PDLS (n=3) or cultured in conventional 2D were analyzed by RNAseq. Uniform approximation and projection (UMAP) plot is shown. **(B)** Volcano plot representing the differentially expressed genes (DEG) comparing purified B cells from FL-PDLS or cultured in 2D (left panel). Heatmaps of DEG for the individual patients (n=3) (right panel). NS: non-significant; Down: Downregulated; Up: upregulated. **(C)** Gene Set Enrichment Analysis: PDLS vs 2D samples. The ridgeplot visualizes the expression distributions of core enriched genes for selected GSEA enriched gene sets. Gradient color reflects the p-values (left panel). Corresponding enrichment plots are also shown (right panel).

Figure S4

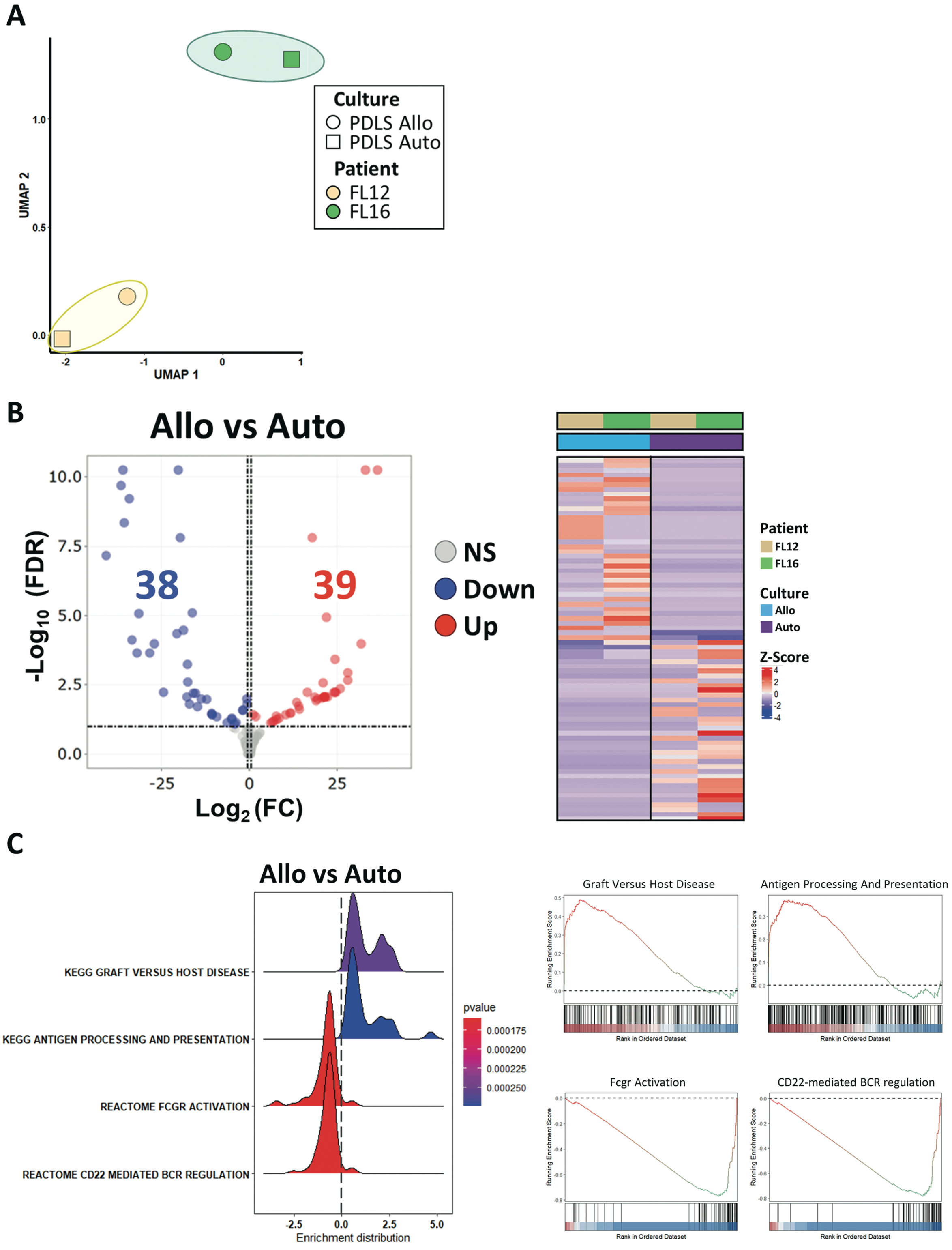

Figure S4. Comparison of FL-PDLS generated with allogenic or autologous monocytes. (A) Purified B cells from FL-PDLS (n=2) generated with allogenic or autologous monocytes were analyzed by RNAseq. Uniform approximation and projection (UMAP) plot is displayed. **(B)** Volcano plot representing the differentially expressed genes (DEG) comparing PDLS with allogenic monocytes (Allo) or containing autologous monocytes (Auto) (left panel). Heatmaps of DEG for the individual patients (n=2) (right panel). NS: non-significant; Down: Downregulated; Up: upregulated. **(C)** GSEA analysis ridgeplot of top 4 significant-enriched GO pathway gene sets. Corresponding enrichment plots are also shown (right panel).

Figure S5

A

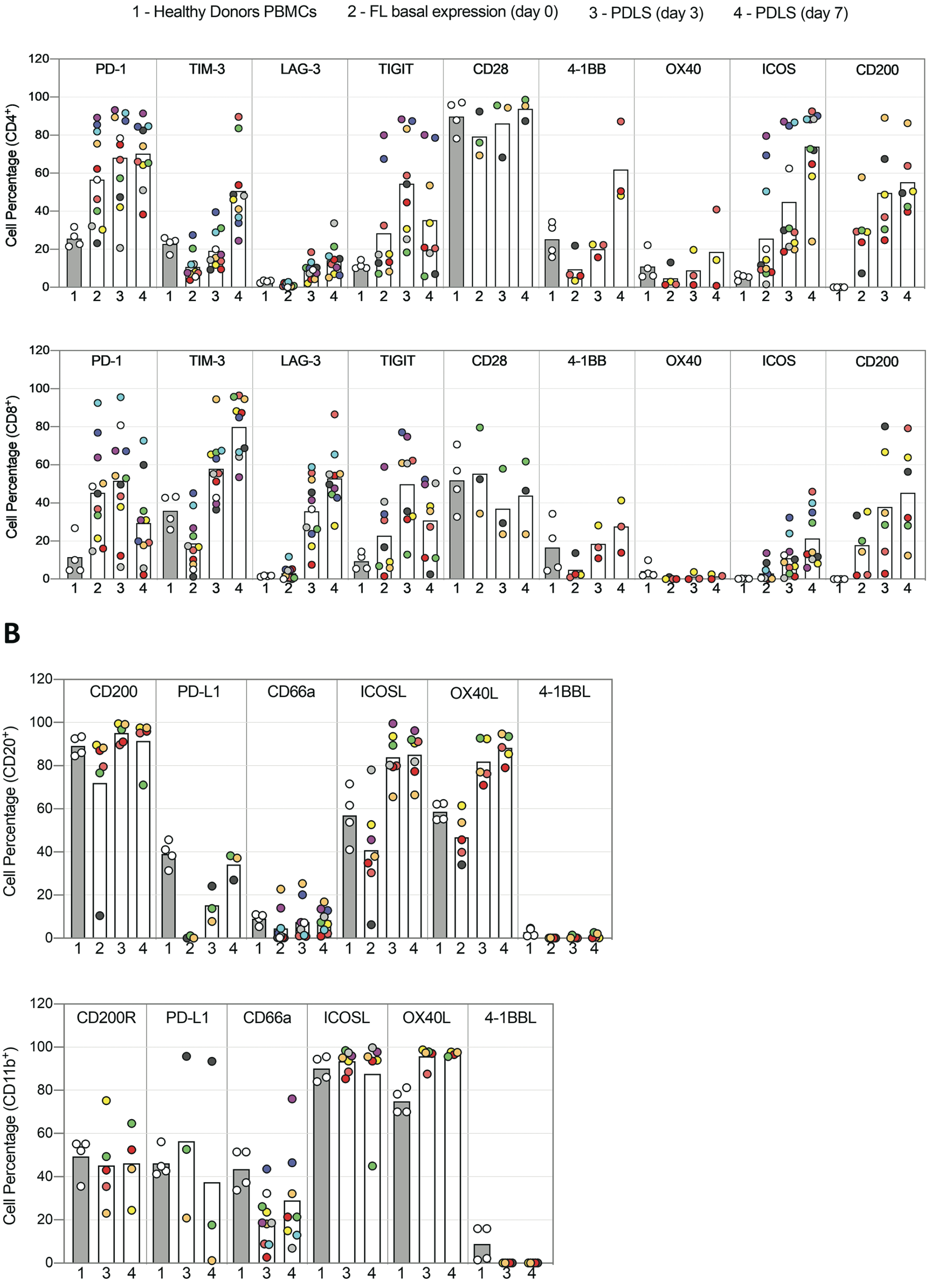

Figure S5. Immune regulators expression in autologous T cells from FL-PDLS. (A) Graph bars showing mean percentages of IC expression on CD4 and CD8 T cells, or their ligands in CD20 and CD11b cells **(B)** from (2) baseline (day 0), (3) day 3, and (4) day 7-FL-PDLS. Control expression is measured in healthy donors PBMCs (1). Each colored dot represents a FL-PDLS patient.

Figure S6

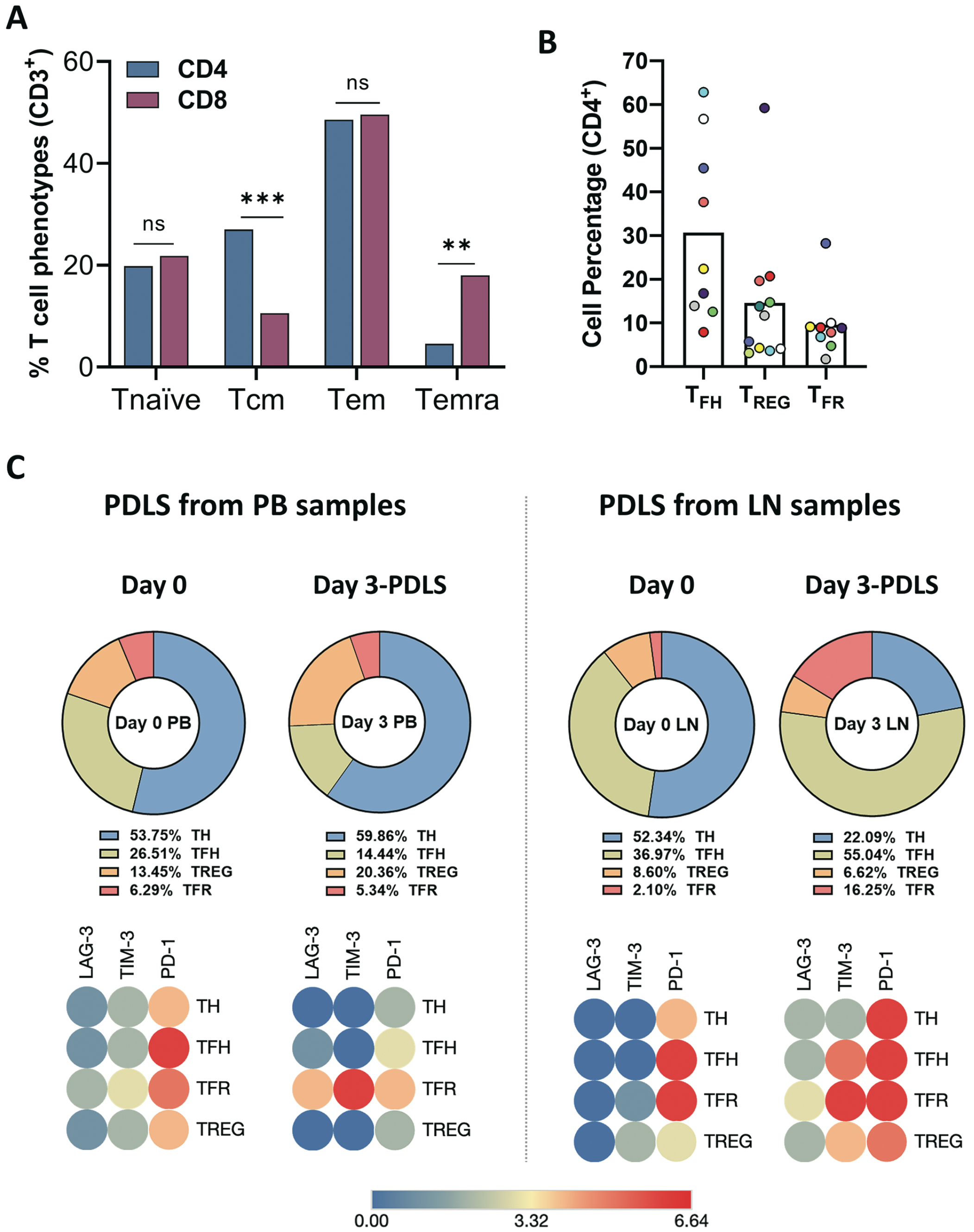

Figure S6. T cell phenotypes present in FL-PDLS. (A) Mean percentage of each CD4^{+} or CD8^{+} phenotype for FL patient measured by CD45RA and CCR7 expression by flow cytometry at day 3-PDLS. Paired t test - Wilcoxon matched-pairs signed rank test was applied. **(B)** Cell percentage of T_{FH}, T_{REG} and T_{FR} measured by FoxP3 and CXCR5 expression by flow cytometry at day 3-PDLS within the CD4 population (as in figure 3F) including patient coding**. (C) C**ell percentage of T_{H}, T_{FH}, T_{REG} and T_{FR} measured by FoxP3 and CXCR5 expression by flow cytometry at day 0 and day 3-PDLS generated from PB or LN samples. The relative expression of LAG-3, TIM-3 and PD-1 is displayed in these four conditions.

Figure S7

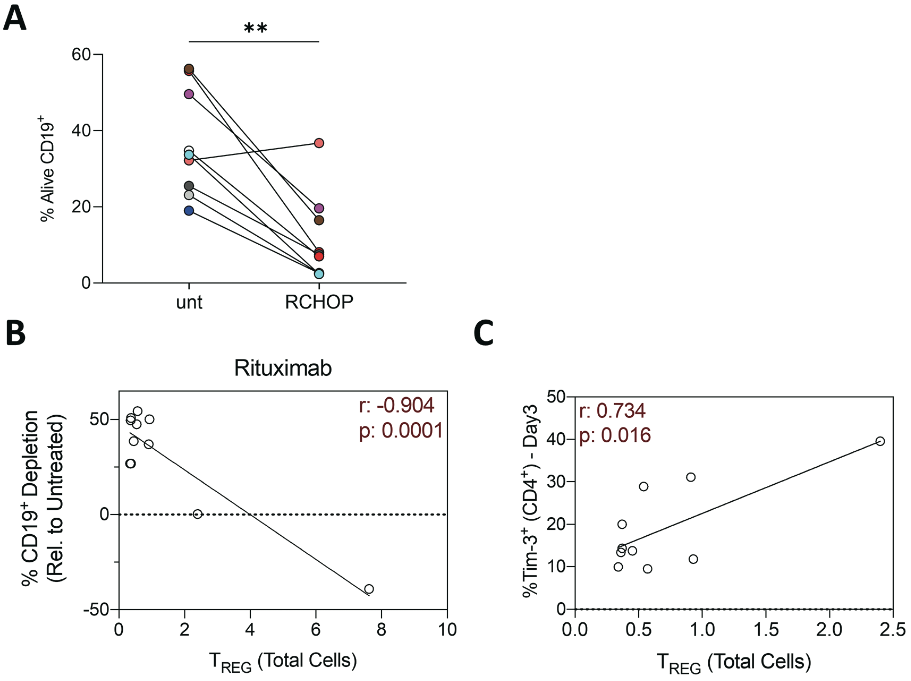

Figure S7. TIM3 expression and rituximab activity. (A) Day 3 FL-PDLS were treated with R-CHOP or remained untreated for 3 days; the percentage of alive B cells is represented. Paired t test - Wilcoxon matched-pairs signed rank test was applied. **(B)** Correlation plot (simple linear regression) between percentage of total T_{REG} in the autologous day 3-FL-PDLS T population and rituximab depletion. **(C)** Correlation plot (simple linear regression) between TIM-3 expression at day 3 in $CD4^+$ cells and the percentage of T_{REG} cells out of total alive cells from the FL-PDLS.

Figure S8

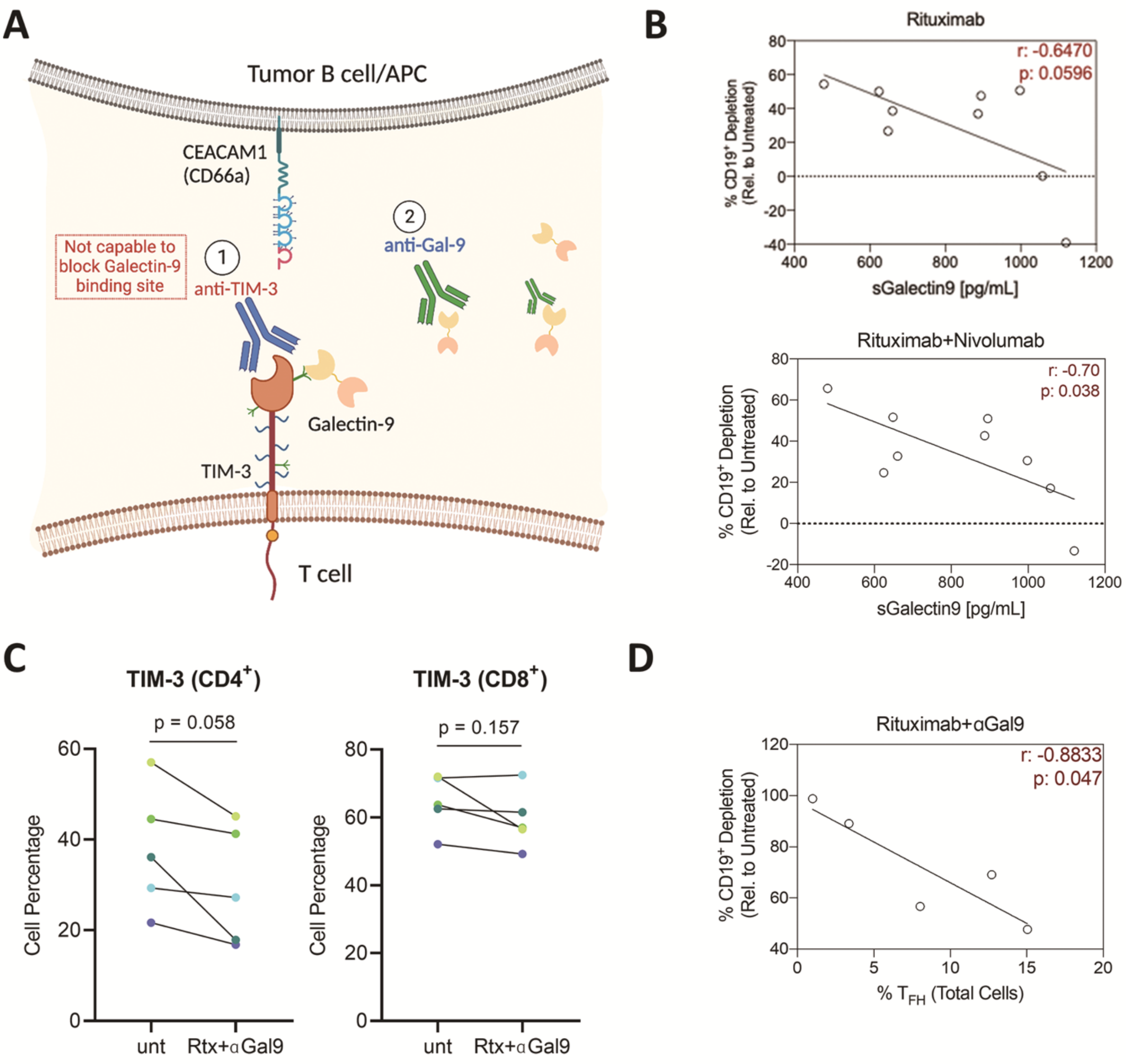

Figure S8. Galectin-9 is a novel target modulating TIM-3 expression. A) Schematic representation of receptor TIM-3 and CD66a and galectin-9 ligands and how blocking antibodies interact. Created with BioRender.com. **(B)** Correlation plot (simple linear regression) of tumor B cell depletion in FL-PDLS treated with rituximab (upper panel) or rituximab + nivolumab (lower panel) and the concentration of soluble galectin-9 in the supernatants. **(C)** TIM-3 expression in CD4 and CD8 cells in FL-PDLS untreated (unt) or treated with rituximab + mAb anti-galectin- 9 (Rtx+Gal9). Paired t test - Wilcoxon matched-pairs signed rank test was applied. **(D)** Correlation plot (simple linear regression) of tumor B cell depletion in FL-PDLS treated with rituximab + anti-galectin-9 and the percentage of T_{FH} out of total alive cells from the PDLS.

SUPPLEMENTAL TABLES

Table S1. Antibodies used in flow cytometry, immunofluorescence and functional assays

Table S2. Genes from LN signatures

Table S3. Common deregulated genes in LN vs PB and PDLS vs PB comparatives

Tables are available as individual pdf files.
