# Supplementary material for: Patient-derived follicular lymphoma spheroids recapitulate lymph node signaling and immune profile uncovering galectin-9 as a novel immunotherapeutic target
Source: Blood Cancer J. 2024 May 2;14(1):75. doi: 10.1038/s41408-024-01041-7 (PMC11636880; doi:10.1038/s41408-024-01041-7)
Supplement: Supplementary file 4 — Table S1 [file 41408_2024_1041_MOESM4_ESM.pdf]

Table S1. Antibodies used in flow cytometry, immunofluorescence and functional assays

Target	Conjugate	Clone	Dilution
CD19	Unconjugated	6OMP31	1/100
CD3	Unconjugated	Polyclonal	1/100
Ki-67	Unconjugated	MIB-1	1/100
Goat anti-Rabbit	AF405	Polyclonal	1/500
Goat anti-Mouse	AF488	Polyclonal	1/500
Goat anti-Rat	AF568	Polyclonal	1/500
CD19	PE	SJ25C1	1/200
CD20	eFluor 450	2H7	1/50
CD3	Pe-Cy5	UCHT1	1.5/100
CD4	SB600	RPA-T4	1.5/100
CD66a	AF488	283340	1/50
CD8	APC-H7	SK1	1/100
CD8	SB600	RPA-T8	1.5/100
PD-1	PE-Cy7	EH12.1	1/50
PD-L1	PE	MIH1	1/200
TIGIT	APC	MBSA43	1/50
TIM-3	PE	F38-2E2	1/50
CD3	FITC	SK7	1/50
FOXP3	PerCP-Cy5.5	PCH101	1/20
CXCR5	FITC	RF8B2	1/200
CCR7	PE	3D12	2.5/100
CD45RA	PE-Cy7	L48	1/100
CD3	AF700	SP34-2	1/50
CD11b	PerCP-eF710	ICRF4A	1/50
CD28	FITC	CD28.2	1/5
LAG3	APC	3DS223H	1/50
PD-1	BV605	EH12.1	1.5/100
ICOS	Pe-Cy7	ISA-3	1/100
41BB	SB436	4B4-1	1/50
OX40	PE	ACT35	1/50
CD200	PE	OX104	1/50
ICOSL	SB600	2D3/B7-H2	1/50
OX40L	PE	11C3.1	1/50
41BBL	PE-Cy7	4H3	1/50
CD200R	APC	OX-108	1/100
FUNCTIONAL GRADE ANTIBODIES			
anti-ICOS	Unconjugated	ISA-3	1/100
anti-TIM-3	Unconjugated	F38-2E2	1/200
anti-LAG-3	Unconjugated	17B4	1/500
anti-TIGIT	Unconjugated	MBSA43	1/100
anti-Galectin-9	Unconjugated	9M1-3	1/200
ISOTYPE CONTROLS (functional experiments)			
anti-IgG1, kappa	Unconjugated	P3.6.2.8.1	1/100
