# Supplementary material for: Patient-derived follicular lymphoma spheroids recapitulate lymph node signaling and immune profile uncovering galectin-9 as a novel immunotherapeutic target
Source: Blood Cancer J. 2024 May 2;14(1):75. doi: 10.1038/s41408-024-01041-7 (PMC11636880; doi:10.1038/s41408-024-01041-7)
Supplement: Supplementary file 5 — table S3 [file 41408_2024_1041_MOESM5_ESM.pdf]

Table S3. Common deregulated genes in LN vs PB and PDLS vs PB comparatives

Common upregulated genes in LN vs. PB and PDLS vs. PB comparatives:

Description	Log2 PDLS vs PB	pval PDLSvsPB	Log2 LN vs PB	pval LNvsPB
IGLV1-40	10.4057398	0.089455312	2.554588852	0.806414141
IGLV6-57	9.380860987	0.066956498	2.771058793	0.465602312
WNT11	8.766896943	0.041732188	2.760812336	0.298761969
CDC45	7.522064726	0.019796279	2.792018155	0.14132552
IQGAP3	7.191499907	0.007210572	3.62309763	0.043240896
XIRP1	7.112625673	0.094493798	3.158429363	0.159023616
PKMYT1	7.092197223	0.008623562	2.69517913	0.078361888
IGLV3-10	7.076275067	0.546503806	2.295748443	0.817426908
ATP23	6.965784285	0.735655974	3.770073906	0.876166059
MND1	6.956811732	0.000650325	2.812498225	0.132304285
CPLX1	6.932847467	0.196280405	4.896548651	0.232857938
WNT5B	6.776981273	0.019143397	3.173260714	0.42189989
KIFC1	6.665901325	0.088796668	2.161649639	0.313321446
SLC16A9	6.62935662	0.059489482	4.321928095	0.095416929
JCHAIN	6.574290816	0.033744063	2.307932002	0.435291316
ACOT4	6.547408698	0.129119704	2.874469118	0.499079133
TIMM22	6.447409542	0	6.436190995	0
FAM50B	6.438098749	0.002240609	2.101538026	0.080069989
ZNF77	6.407692649	0.450370062	2.277603334	0.786076861
SLC9A9	6.348074247	0.370957635	2.561316878	0.702664158
E2F8	6.293083591	0.002847465	2.391669166	0.02609497
SPNS3	6.291554446	0.016583529	2.36923381	0.232102695
H2BC7	6.262094845	0.086768472	4.491853096	0.291212847
LDHAP4	6.254241287	0.017461029	3.029747343	0.082586252
E2F7	6.085251136	0.001168436	3.495588577	0.030576143
LSM2	6.072913054	0.379006074	3.142823192	0.54965255
BCAS2P2	6.055282436	0.068358054	2.584962501	0.037749551
ZNF618	5.940837928	0.010932664	2.335603032	0.616680991
VDR	5.818207394	0.010524957	2.892561835	0.402079364
ANLN	5.814764979	0.001547801	2.59647959	0.004697776
HNRNPA1P70	5.807354922	0.027016589	3.523561956	0.042771032
SCML1	5.74819285	0.042979124	2.392317423	0.1126099
ARRDC4	5.721099189	0.018249236	3.247927513	0.024734262
CCL17	5.683495955	0.091952978	4.093511886	0.106416144
CEBPA	5.617073842	0.379627341	3.061467633	0.43342009
LEF1	5.575490763	0.046729868	3.142957954	0.085542632
H1-3	5.569855608	0.046529335	7.45532722	0.332266521
ARHGEF25	5.541638734	0.305339749	3.319039816	0.463235404
NEIL3	5.496311684	0.024581064	2.394932186	0.424752081
ATP9A	5.47045552	0.011380786	2.008988783	0.052038154
MSL3P1	5.427662038	0.197810886	2.110845769	0.631814261
DNMT3B	5.388755363	0.030005468	2.078138097	0.140588375
H2AC19	5.384801885	0.371351199	5.632708877	0.308884968
SLC7A5	5.339126765	0.21051048	2.96853075	0.369855754
GNB3	5.244231678	0.013642377	3.683384554	0.347574394
VEGFA	5.22502481	0.445093344	2.045388581	0.672180996
CHST2	5.217851289	0.098316579	2.303862313	0.536765527
POU4F1	5.087462841	0.072680049	5.053111336	0.525052946
SYNGR3	5.064535511	0.137223807	2.711733148	0.332229363
SH3RF1	5.027905997	0.001754131	2.309855263	0.027211613
NUSAP1	5.025730051	0.003286401	2.025778441	0.042996304
TRIB1	4.987162285	0.21913123	3.651083805	0.312818256
IGSF3	4.941862734	0.180870599	3.255290902	0.173681211
IGKJ5	4.903132663	0.009633189	2.018016143	0.109588964
EPHB1	4.87677487	0.225169626	3.381161202	0.604510274
ARHGAP19-SLIT1	4.87583466	0.059198395	3.106199404	0.718825229
SDC4	4.871492409	0.020280648	2.060856874	0.414947311
GLUD1P2	4.866248611	0.2709437	2.115477217	0.44384996
SVIL2P	4.865423978	0.019033034	3.439623138	0.271415748
CDC42EP4	4.748640128	0.0923171	2.468148836	0.412876933
FOSL2	4.659054942	0.040431207	3.293016242	0.078802121
H2AC8	4.618385502	0.099328951	4.456012098	0.389060926
MYBL2	4.539023883	0.059575866	2.464247891	0.231125526
DUSP4	4.537176307	0.114142583	3.661241245	0.219196248
SOCS3	4.534394641	0.014092752	2.082424011	0.060666109
PDGFD	4.521783627	0.453485599	2.343407822	0.581160714
PLXNB2	4.507829561	0.075249297	2.366234326	0.326730295
DRD2	4.503825738	0.496345605	2.330916878	0.908175784
PROSER2	4.491853096	0.034421031	2.906890596	0.029989053
LSM2	4.473931188	0.379006074	3.713481985	0.54965255
MT2A	4.452941161	0.019645852	8.118758363	0.230194723
SPR	4.442943496	0.013710603	3.781359714	0.018534802

Common downregulated genes in LN vs. PB and PDLS vs. PB comparatives:

Description	Log2 PDLS vs PB	pval PDLSvsPB	Log2 LN vs PB	pval LNvsPB
MCF2	-2.06082184	0.088482514	-2.117463838	0.200686177
ROPN1	-2.125530882	0.221507671	-2.125530882	0.221507671
ADAMTS18	-2.263034406	0.015890433	-2.263034406	0.015890433
ARL13A	-2.317926164	0.243552123	-2.255360478	0.022562021
VN1R51P	-2.321928095	0	-2.321928095	0
HIPK4	-2.415037499	0.019963367	-3.263034406	0.025985231
RPS18P13	-2.469485283	0.047583757	-2.08246216	0.113498442
RPL13AP7	-2.872244453	0.006018249	-2.388565288	0.046394601
TACR1	-2.876193798	0.063560434	-2.31676639	0.164868038
BNIP3P5	-2.938236832	0.087117103	-2.233313708	0.234872177
EPHB6	-2.957042556	0.066862585	-2.409519619	0.195694438
PGLYRP1	-3.037474705	0.062728898	-2.142444265	0.020719729
CHORDC1P1	-3.063193826	0.170777626	-3.063193826	0.170777626
DUX4L26	-3.459431619	0.039330896	-3.874469118	0.015575239
ARSJ	-3.463400521	0.043271382	-3.463400521	0.043271382
CA15P1	-3.588418059	0.034033068	-2.303636946	0.323541892
FAM246C	-3.660993057	0.026740156	-2.471987185	0.212888754
RWDD4P1	-3.698002439	0.003123441	-2.046748259	0.004132574
GLYATL1B	-3.773202456	0.100938791	-2.144801957	0.077775343
ACSM3	-3.91270099	0.04616299	-3.225476672	0.067533033
FOXE1	-3.948367232	0.004711027	-2.456514135	0.049196228
EPHB6	-4.04580369	0.066862585	-3.009423254	0.195694438
YBX2P1	-4.191224403	0.034993049	-2.732825864	0.141723844
NXF5	-4.321928095	0.034724178	-2.065588342	0.117393854
ABLIM2	-4.876042469	0.023222113	-2.967262062	0.029536949
ACSM1	-5.088851533	0.047932532	-3.161349746	0.023764318
CYP2A6	-5.617601317	0.035224888	-2.130865131	0.215321121
IL24	-5.922617097	0.008805286	-2.580358912	0.027219418

INA	4.437405312	0.080770425	5.392317423	0.09536852
SRGN	4.425339846	0.010973964	2.319157422	0.269993346
H2BC11	4.423942162	0.00297498	3.535659581	0.015206083
CDCA2	4.391023352	0.028683674	2.066458162	0.194054571
GPI	4.374884228	0.00384754	2.611024797	0.899562137
TAGLN3	4.339850003	0.015877242	2	0.063453021
CXCL8	4.303780748	0.026624591	3.176588732	0.014805639
H2BC17	4.297680549	0.177160989	3	0.207898942
PTGER1	4.285402219	0.006315636	3.584962501	0.422649731
H2AC11	4.273018494	0.030123797	4.087462841	0.087454273
HSPA1L	4.271463028	0	2.475733431	0
CDR2L	4.255500733	0.081438539	2	0.347371582
PALD1	4.25244017	0.591119346	2.790313399	0.495926693
SMIM1	4.235216462	0.13257491	2.700439718	0.087457619
OXCT2	4.230100516	0.013989184	2.153805336	0.335468602
KCNN3	4.189142917	0.012019567	2.189688256	0.534587335
RNF208	4.153024827	0.174847364	2.10319005	0.326972192
TNFRSF17	4.137884735	0.02294272	2.871797813	0.145946541
PRSS36	4.129283017	0.000902343	2.700439718	0.211480963
KIF7	4.125450372	0.160548622	2.064242344	0.495704387
WHAMMP2	4.111801766	0.602861143	3.738285199	0.536493445
CADM1	4.07516731	0.043988622	2.661989281	0.402664508
EBF4	4.058237769	0.518313589	2.666412314	0.676966772
SMIM24	4.05166212	0.168996289	3.115477217	0.597684477
SLC7A5P1	4.042902962	0.416984309	2.421682342	0.545383571
TNF	4.036309919	0.472367803	4.525015508	0.902859799
FRMD6	4.028826964	0.276826813	3.341333494	0.40732708
TNFRSF11A	4.027516566	0.024993332	2.094532931	0.090035348
MCCC2	3.931988157	0.611954473	2.83516482	0.662595109
H2AC15	3.918863237	0.641431836	4.536247216	0.510586603
H2AC7	3.912889336	0.114683184	3.294620749	0.405076014
IL1B	3.898853277	0.133827341	2.969626351	0.409725094
MCCC2	3.898853277	0.611954473	2.399930607	0.662595109
H4C4	3.857980995	0.066986629	3.584962501	0.019803941
PRR3	3.83385959	0.786134698	3.065014978	0.978030775
SERP2	3.776981273	0.01410952	3.094517599	0.674690942
H1-4	3.776103988	0.072089868	6.477353527	0.150583955
MTARC2	3.772589504	0.065351459	2.807354922	0.134235847
RPP21	3.77149969	0	4.076120633	0
H2AC16	3.745954377	0.023875624	3.653442239	0.235009824
CD27	3.703494731	0.016796759	2.52142835	0.191901916
HSP90AA2P	3.700439718	0.003669521	4.392317423	0.002239817
ELL2	3.669126113	0.019679856	2.797264782	0.076790054
ANKRD36BP2	3.658012956	0.515396947	3.57821019	0.285121775
GNG2	3.611847321	0.034110333	2.057246903	0.362559848
NAV2	3.576979638	0.198892963	3.130396637	0.4853104
SEMA4A	3.557876399	0.326012329	2.949131825	0.493025246
CTNNAL1	3.497150691	0.275827502	2.123813692	0.381976069
H2AC12	3.442943496	0.354242889	3.288042101	0.689723135
NECTIN1	3.422568654	0.199102207	4.084320966	0.227872198
KCNQ1	3.398549376	0.246671609	3.184424571	0.339688357
H1-2	3.383137394	0.023895048	4.017404795	0.229329747
P4HA2	3.3682332	0.022414238	3.109564782	0.396886021
GTF2H4	3.359021801	0.422656113	2.991641911	0.795797833
ZBTB9	3.357552005	0.502663985	2.280107919	0.554096175
SPSB1	3.342832743	0.321579593	2.104779463	0.465162474
HLA-L	3.322093532	0	4.460258615	0
MT1X	3.314749831	0.017533836	8.031981557	0.19746185
TCEAL9	3.275842326	0.30363272	4.122082831	0.25390529
LGALS3	3.269392329	0.083285154	2.134469587	0.216606328
DLX4	3.263034406	0.255548546	2.655351829	0.24091036
RBM11	3.247927513	0.359103882	2.295455884	0.406466159
MYOM2	3.215349144	0.154848596	2.352210662	0.165167537
SLC30A1	3.19172101	0.533217994	8.22764711	0.249593983
FGF2	3.174370906	0.201722643	2.959358016	0.203914666
H2AC13	3.156504486	0.099848666	3.849665727	0.515440646
DEPP1	3.116863758	0.042309521	2.624490865	0.195739796
HLA-DQB1	3.099938068	0	2.54778652	0
C1orf194	3.022367813	0.239341369	2.672425342	0.179704893
H2AC20	3.004501392	0.212657768	3.334695391	0.426054751
FOSL1	2.995129765	0.050329733	3.135063753	0.087401284
IFFO2	2.979206023	0.02606318	3.316821453	0.292914442
ULBP1	2.92071675	0.242062634	2.680574412	0.257690762
KLF4	2.906890596	0.100395232	3.502500341	0.088205431
NPW	2.906890596	0.064612673	2.584962501	0

H2BC4	2.881269626	0.071579436	3.868503094	0.228920927
LAP3P2	2.816692787	0.314744981	2.483082887	0.413593969
MT1F	2.816110937	0.076504965	6.769048901	0.231958059
MYL2	2.772589504	0.103438071	2.736965594	0.651680505
SLC5A3	2.750969498	0.333090124	2.618677733	0.203924954
PINX1	2.747745433	0.191978725	2.011972642	0.778842
DDAH2	2.686440923	0.844400153	3.08239821	0.635910882
H4C2	2.678071905	0.257559196	3.217230716	0.132810228
FCGR1A	2.662965013	0.16441885	2.459431619	0.309152659
DLL1	2.637161344	0.452872534	2.798064682	0.273992699
DNAJA4	2.608939534	0.739969004	2.628090611	0.714986705
HNRNPA1P36	2.590416931	0.898165661	2.851181163	0.682243116
ATRNL1	2.584962501	0.332513207	2.087462841	0.323358207
HSPA1B	2.57790312	0	8.208344755	0
ZNRF2P2	2.562936194	0.746328133	2.035090664	0.696510659
HSPA1A	2.516096978	0	6.5188258	0
ETS2	2.49546547	0.116334209	2.773168602	0.212885208
SPAG17	2.483517237	0.853150253	2.772589504	0.581295954
SMKR1	2.476813697	0.024573072	2.321928095	0.338640153
HEY1	2.433986602	0.128682917	2.103691303	0.166946244
STIP1	2.423171954	0.093269981	2.271218098	0.102298572
RASSF6	2.400263507	0.947492777	3.369370421	0.286366797
SMAD7	2.398291762	0.91097556	2.83103511	0.648062771
FAM156B	2.394681044	0.417960345	2.295904284	0.219148931
EGFLAM	2.36923381	0.300603521	2.564784619	0.375160526
MALLP2	2.363658659	0.220349148	4.342116089	0.307432727
RAP1BL	2.321928095	0.422649731	2.169925001	0.082255771
OR5B21	2.294385111	0.934978014	2.277794053	0.763187041
CMTM8	2.247927513	0.186546398	2.280107919	0.443481748
RELL1	2.214487665	0.440803609	2.527343293	0.34838906
KHDRBS3	2.207892852	0.862494785	3.710393192	0.487075485
MRPL18	2.203185197	0.011201768	2.350541248	0.113064476
SEMA3G	2.199672345	0.876881709	2.238404739	0.483623143
COL5A1	2.187627003	0.382068568	2.256339753	0.82241434
KCNIP4	2.180937994	0.908937043	2.617752436	0.75785777
CACYBP	2.174330233	0.057977257	2.281278991	0.052499893
PPT2	2.142019005	0	2.117866973	0.739202338
OR2T2	2.115477217	0.164368425	2.502500341	0.745869347
HSPD1	2.110514885	0.04981584	4.192293395	0.013172474
C15orf65	2.102810806	0.946657874	2.517848305	0.654642526
LTBP1	2.079229328	0.522873124	2.729171437	0.279646471
COQ10BP2	2.070389328	0.638924926	2.201633861	0.300727417
NINJ1	2.069705246	0.043136274	2.466555989	0.448768502
SLC43A1	2.065965641	0.513640038	2.581563441	0.397701644
RRM2P3	2.058893689	0.211741771	2.222392421	0.225750902
LMNA	2.052394927	0.943663069	3.779563933	0.530044982
WNT10A	2.030104903	0.041974939	2.161028155	0.613528479
MTTP	2	0.673755977	2.754887502	0.623632156
