# Supplementary material for: Patient-derived follicular lymphoma spheroids recapitulate lymph node signaling and immune profile uncovering galectin-9 as a novel immunotherapeutic target
Source: Blood Cancer J. 2024 May 2;14(1):75. doi: 10.1038/s41408-024-01041-7 (PMC11636880; doi:10.1038/s41408-024-01041-7)
Supplement: Supplementary file 6 — Table S2 [file 41408_2024_1041_MOESM6_ESM.pdf]

Table S2. Genes from LN signatures.

Upregulated genes in LN:

Gene Symbol	log2FoldChange	pvalue	padj
ABCA1	1.229398955	0.000774369	0.026582489
ABCG1	1.829684528	2.85884E-06	0.000376865
ABHD3	1.484835006	7.98946E-07	0.000138339
ABI1	0.667771913	9.60198E-06	0.001007674
ABI3	0.522447145	0.004380407	0.087654549
ACP3	1.030754326	0.000384449	0.015996143
ACSL1	0.760309211	0.00074212	0.025864646
ACSL3	0.840405847	0.000103742	0.006379926
ACY3	1.336352657	0.000528537	0.020280888
ADAM8	1.004347429	3.36275E-08	1.04135E-05
ADGRG5	0.894803349	0.00070436	0.024823417
AHSA1	1.733333903	1.87541E-08	6.81278E-06
ALG2	0.589477753	0.001401862	0.040615295
AMZ2P1	0.722953251	0.000884281	0.029000106
ANKRD13A	0.667304755	0.000283073	0.013010345
ANKRD28	0.879857747	0.00108736	0.034119991
ANKRD36BP2	0.617924721	0.005398513	0.099103894
ANLN	2.538016795	3.25545E-10	1.75701E-07
ARG2	1.026597517	0.000364891	0.015366938
ARHGAP29	0.665515629	0.000221827	0.010912264
ARHGEF11	0.64776361	0.003619519	0.07805104
ARIH1	0.880582975	4.07496E-05	0.003103874
ARRDC3	0.692476139	0.002892334	0.06695612
ARRDC4	0.799928214	0.002533405	0.061196947
ASF1B	1.413984379	2.78269E-06	0.000372801
ASPM	1.60203353	3.95358E-06	0.000475689
ATF3	2.383235176	3.15489E-08	1.0101E-05
ATF5	0.615338798	0.005306496	0.098564114
ATP1A1	0.603856176	0.000568406	0.021474393
ATP1B3	0.707508759	0.000468079	0.018693461
ATP9A	1.383330025	0.000241945	0.011599845
AZIN1	1.160531159	1.15087E-05	0.001151909
B3GNT7	0.635595206	0.003904577	0.08115674
BAG3	3.343647811	7.71343E-12	6.62303E-09
BAK1	0.891231731	0.00049028	0.01925445
BBC3	0.630291506	0.003131237	0.070248296
BCL2L11	2.427963644	4.38587E-11	2.76163E-08
BCL2L2-PABPN1	0.626162674	0.002657526	0.063304758
BCL3	1.087040288	0.000104024	0.006379926
BRI3BP	0.629771162	0.000875236	0.02885378
BTG3	1.466307612	6.69271E-06	0.000748078
BUB1	1.07825768	3.23682E-06	0.000418792
C12orf75	1.376478196	1.17954E-05	0.001172707
CAB39L	0.580046112	0.001428821	0.041081331
CACYBP	2.321145142	1.41523E-17	6.68341E-14
CADM1	0.962664555	0.001584498	0.044211473
CALM1	0.881503186	0.000236937	0.011446926
CALU	0.655335024	0.001493471	0.042359863
CAMK4	0.71576224	0.003732467	0.079758257
CBX4	1.308938172	3.48613E-07	6.9319E-05
CCDC106	0.770370588	0.001531923	0.043255657
CCDC110	1.406100249	0.000530049	0.020280888
CCDC117	1.458357681	2.52517E-07	5.4828E-05
CCDC6	1.345164427	1.03195E-05	0.001065225
CCNE1	0.549634479	0.003788182	0.080222824
CCNL2P1	1.72022861	4.66177E-05	0.003426493
CCNYL1	0.628376931	0.000348187	0.014903062
CCT2	0.602205212	0.000331758	0.014394246
CCT3	0.629954767	0.001326357	0.039292039
CD101	1.398855443	0.000503912	0.019626592
CD27	1.844859529	2.9337E-05	0.002419986
CD81	0.763155433	0.000319577	0.01417092
CDC20	0.788727085	0.002334456	0.058023517
CDC42EP4	2.132058812	4.68624E-05	0.003427664

Downregulated genes in LN:

Gene Symbol	log2FoldChange	pvalue	padj
ABHD16B	-0.626804203	0.00043229	0.01741159
ACSM3	-2.181118874	6.0386E-08	1.7283E-05
ADAM20P1	-0.978412176	0.00121872	0.03716695
ADAMTS7P3	-1.978574545	0.00013358	0.00757734
ADGRG3	-0.622932345	0.00427752	0.08632012
ADGRL1	-1.877685559	4.3139E-09	1.8109E-06
AGPAT1	-1.784285032	0.00012989	0.00741259
ALMS1	-0.533744372	0.00121988	0.03716695
ALMS1P1	-0.685968065	0.00150486	0.04255518
ALX3	-0.57730771	0.00331347	0.07303563
ANXA1	-3.008721854	1.3757E-07	3.2895E-05
APBB1	-0.787929494	0.00036312	0.01536694
APLF	-0.544371305	0.00186575	0.04974632
ARAP3	-1.145729512	0.00099888	0.03165925
ARR3	-0.868718698	0.00220297	0.05541171
ARRB2	-0.84714575	8.3793E-05	0.00538383
ARRDC2	-0.680817959	1.4832E-05	0.00140795
ATP6V0D2	-0.826362515	0.00077538	0.02658249
ATP8B4	-2.534789382	9.4599E-07	0.00015955
AVPR2	-0.979685771	0.00036857	0.01543745
BACE1	-0.67004357	0.0026715	0.06355737
BOK	-4.279496446	2.7968E-08	9.4343E-06
BTBD3	-0.670928803	0.00432927	0.08690741
C11orf21	-0.760552068	0.0009199	0.02985715
C1orf216	-0.500949647	0.0008672	0.02873931
CAMK1D	-1.506125712	3.7063E-05	0.00290508
CARNS1	-0.938604462	0.00159594	0.04446497
CBR3	-0.65139628	0.00392894	0.08137903
CCR1	-2.19387505	3.4476E-05	0.00274792
CCR2	-0.667790853	0.00423771	0.08570697
CCT8P1	-0.976902109	0.00091168	0.02964122
CD2	-2.664715863	2.7495E-06	0.0003728
CD7	-2.183585287	1.5943E-05	0.0014909
CDHR2	-1.891699146	0.0001285	0.00740037
CDHR5	-0.719997297	0.00348622	0.07595689
CDRT15P1	-1.277384772	0.00031878	0.01416871
CHRM5	-0.500104412	0.00537482	0.09895748
CIPC	-0.583886318	0.00175054	0.04771673
CISH	-0.967677414	0.00075623	0.02621121
CLEC16A	-0.508355985	0.00060014	0.02218745
CLEC18A	-1.197669847	7.3532E-07	0.00012981
CLTCL1	-0.819881366	0.00122552	0.03721869
CPNE1	-0.781499575	0.00010547	0.00642688
CRIM1	-1.284293718	0.00011173	0.00667194
CROCCP2	-0.500099134	0.00070678	0.02486223
CSF1	-1.26066825	0.00014332	0.00797309
CST7	-1.614672568	0.00012678	0.00732368
CXCR3	-0.841210884	0.00222366	0.05577205
DDTL	-9.318746785	3.1661E-05	0.00257788
DNAAF9	-0.635890644	0.00192715	0.05068777
DNMT3A	-1.31110186	0.00015723	0.00848601
DOCK11	-0.797857573	2.0019E-05	0.00177814
DPEP2	-1.182612735	9.2154E-05	0.00582467
DUS4L-BCAP29	-0.686014656	0.00066712	0.02382204
DUSP22	-0.588195916	0.00029423	0.01344897
EEF2KMT	-0.823334321	0.00109926	0.03420939
EFCAB8	-1.196785818	0.00085755	0.02852033
EMILIN1	-1.084035012	0.00019461	0.00993553
EPHB6	-1.690310139	0.00012248	0.00720775
ERP27	-0.692927865	0.00126862	0.03815963
FAAH	-1.166677934	0.00021062	0.01048756
FAM111B	-0.879048549	5.2215E-05	0.00372201
FAM171A1	-0.547669543	0.0037314	0.07975826
FAM246C	-0.793876561	0.00274007	0.064298

CDC45	1.076655549	0.000967689	0.030931809	FAM53B	-0.564382674	0.00012305	0.00721857
CDCA2	2.303683672	1.74284E-12	1.82902E-09	FASLG	-0.622021571	0.00489565	0.09398261
CDCA5	1.224754059	0.000241798	0.011599845	FBXL19	-0.995911461	2.4295E-06	0.00033745
CDCA7	1.288947794	0.000102882	0.006351141	FCGR2C	-0.728560749	0.0020727	0.05323245
CDCA7L	0.559763645	0.00299184	0.068338406	FCGR3A	-2.141635114	1.3679E-05	0.00132511
CDCA8	0.589670205	0.003070316	0.069459002	FCMR	-1.193078074	0.00026105	0.01226689
CDK1	1.760288804	2.00141E-11	1.40024E-08	FGFBP2	-3.939949878	3.7953E-06	0.00046607
CDKN2C	1.428722589	0.000216493	0.010733718	FGR	-0.97864802	0.00095752	0.03076122
CDKN3	2.092159616	4.07695E-08	1.24215E-05	FHL3	-1.059102526	0.00014719	0.00813012
CDT1	0.888153873	0.000953218	0.030699298	FIZ1	-0.974754038	4.4725E-07	8.5339E-05
CEMIP2	0.994993084	0.001307064	0.039005431	FLI1	-0.523053294	0.00062065	0.0226847
CENPE	0.64366154	0.002575504	0.061897292	FOXI1	-0.894113027	0.0020308	0.05233545
CENPF	1.109035332	4.55041E-07	8.59572E-05	FUT7	-1.337611905	0.000573	0.02155226
CFAP20	0.957356238	0.000104754	0.006403884	GATA3	-1.967138611	1.2975E-05	0.00127656
CHDH	1.4031108	0.000195201	0.009938944	GGPS1	-0.54262528	0.00166369	0.04587885
CHORDC1	2.834235068	4.99605E-17	1.57292E-13	GIMAP5	-1.771336665	5.4566E-05	0.00380349
CHST10	0.571428877	0.000284243	0.013032406	GIMAP6	-2.537730418	1.1525E-05	0.00115191
CHST2	1.464023137	0.000342865	0.014753369	GNA15	-1.055947381	0.00118963	0.03648078
CITED2	0.6848171	0.002229111	0.055772053	GNB1L	-0.529337313	0.00378346	0.08022282
CKS2	0.838404102	0.000294752	0.013448971	GNLY	-2.207357136	2.3914E-05	0.00202573
CLGN	0.881376508	0.00193958	0.050816455	GPC1	-0.881136175	0.00133113	0.03929204
COPS8	0.634384893	0.000485556	0.019158692	GPR15	-1.439170399	0.00036785	0.01543745
CPN2	0.800090165	0.000979305	0.031195746	GPRIN1	-0.801395359	0.00074589	0.0259004
CR2	1.512046663	1.06444E-05	0.00109279	GRIN2C	-0.669915836	0.00097627	0.03115174
CREM	1.139253419	0.00014941	0.008228422	GRK4	-0.501763271	0.00138683	0.04042789
CRHBP	1.406305984	0.000467509	0.018693461	GZMB	-3.158610494	1.465E-06	0.00022499
CRY2	0.855881873	0.000270336	0.012577968	HAVCR1	-0.661579872	0.00432447	0.08690347
CRYBA4	0.958409291	0.001011705	0.032011908	HCK	-0.661616734	0.0029769	0.06816189
CRYBB1	4.000811492	9.76934E-06	0.001019574	IGFBP4	-0.801582592	0.00188931	0.04998469
CSRNP1	1.232897859	0.000176569	0.009188417	IGHV3-21	-0.917766652	0.0005946	0.0220667
CSRP1	1.556644476	6.064E-07	0.000110143	IGHV3-64	-19.40418589	7.7028E-06	0.00084341
CTH	1.415101666	8.129E-07	0.000138339	IGHV7-4-1	-0.98966568	0.00053127	0.02028089
CTPS2	0.504757538	0.002590348	0.06209604	IKBKB	-0.509432263	9.5157E-05	0.0059917
CTSO	0.947709968	0.000577546	0.02164652	IL20RB	-0.778783962	0.00124049	0.0374926
CXCL8	2.794404526	3.79962E-06	0.00046607	IL2RB	-1.162438286	0.00025553	0.01211732
CYP51A1	0.706894508	0.000324891	0.014305817	IQCK	-0.522828293	0.0048508	0.09350167
DBF4	0.514069669	0.003138734	0.07033297	KCNJ1	-1.671455323	5.34E-05	0.00375934
DBI	0.543229563	0.003245744	0.0718782	KCNJ11	-0.557300061	0.00512853	0.09682545
DDX12P	0.540807546	0.001867891	0.049746318	KDM8	-0.597727288	0.00179666	0.04855355
DEDD2	1.546578138	1.28426E-06	0.000203863	KIF21B	-0.50720469	0.00460047	0.09080759
DEGS1	0.761443418	0.000762462	0.026282678	KIR2DS2	-1.583913572	0.00025595	0.01211732
DEGS2	1.615912613	0.000137029	0.0077038	KLHL13	-0.823048615	0.00238792	0.05881065
DEPDC1	1.394993974	0.000162171	0.008620878	KRT86	-0.587475028	0.00275052	0.06433926
DHCR7	1.082874817	0.000172766	0.009076329	LBX2	-1.745883346	5.9778E-08	1.7283E-05
DHFRP1	4.116822046	3.66569E-07	7.21301E-05	LCP2	-2.397668005	3.0409E-08	9.9039E-06
DLG5	0.769403824	0.002900421	0.067061143	LIF	-1.717813024	0.00023494	0.01137941
DLGAP5	1.360112331	9.26498E-06	0.000983233	LPAR6	-0.91637459	0.0014762	0.04193307
DLX4	0.695929602	0.003998149	0.082450904	LRFN1	-0.882322528	0.00136695	0.04016497
DNAJA1	0.965664405	0.000232681	0.011328201	LYG1	-0.606508625	0.00501938	0.09552741
DNAJA4	0.718049717	0.002882767	0.066904142	MAF	-0.865010677	0.00115318	0.03553592
DNAJB1	2.690697044	3.44496E-09	1.51338E-06	MAP4	-1.119921233	5.6721E-05	0.00391042
DNAJB4	2.281192652	1.43702E-07	3.35127E-05	MAVS	-0.646824377	2.5525E-06	0.00035195
DNAJB6	2.197047673	5.26515E-14	7.1042E-11	MBD3	-0.538551217	0.00126284	0.03804629
DNAJC19P5	0.875773007	0.001473336	0.041914635	MCF2	-0.591882543	0.00540923	0.0992042
DNMT3B	1.414318712	0.000157215	0.008486009	MCPH1	-1.846924146	2.3553E-05	0.00201595
DPYSL2	0.59629279	0.002692583	0.063817929	MED16	-1.828691076	0.00021097	0.01048756
DSCC1	1.026386243	0.000476357	0.018904187	METTL18	-0.734275546	0.00033175	0.01439425
DSTN	0.644550642	0.00409083	0.08390422	METTL3	-0.551585698	3.1153E-07	6.3964E-05
DUSP10	0.750866618	0.002309928	0.057565348	METTL4	-0.62093867	0.00155903	0.04373151
DUSP2	0.921954086	0.000464798	0.018641249	METTL8	-0.614942435	0.00049244	0.01929899
DUSP4	2.017590117	2.08966E-05	0.001835989	MICB	-20.82712188	1.1493E-11	9.4391E-09
DUSP5	0.920332074	0.00036526	0.015366938	MLC1	-1.756380078	0.00012184	0.00719239
DYRK4	0.529102264	0.002886547	0.066904142	MSH5-SAPCD1	-0.698821928	0.00271099	0.0640133
E2F1	1.080929025	1.13442E-06	0.000184734	MSL1	-0.518230059	0.00015481	0.00848601
E2F7	3.358738872	3.28401E-12	3.10175E-09	MSS51	-1.144777794	9.8452E-07	0.00016314
E2F8	2.280229517	1.53605E-07	3.4959E-05	MUC12	-1.558294069	0.00037552	0.01569384
EFNA5	1.390423461	0.000484503	0.019158692	MUC20	-0.594093629	0.00411528	0.08413155

EGR2	1.21285297	4.31466E-05	0.003196233	MYO18B	-0.64170342	0.00436418	0.08742246
EHD1	1.37513679	1.75357E-08	6.49508E-06	NACC2	-1.208883469	0.00064075	0.02319641
EIF1AD	0.683128196	0.000593905	0.0220667	NCAM1	-1.838045199	5.1743E-05	0.00371429
EIF1AX	0.884124807	2.2376E-05	0.001946906	NCOA6	-0.532921152	0.00047336	0.01882459
EIF1AXP1	1.33091373	2.86528E-06	0.000376865	NEK11	-0.589417813	0.00535832	0.09895748
EIF4H	0.565100342	0.000280513	0.012924144	NEO1	-0.572405274	0.00351263	0.07644413
EIF5A2	0.871612393	0.001382764	0.040427888	NIBAN2	-2.435974677	2.6235E-06	0.00035912
ELL2	2.463001673	1.70769E-06	0.000250063	NKG7	-2.588671229	1.1264E-06	0.00018473
ENC1	0.861897143	0.001331228	0.039292039	NPHP4	-0.653929596	0.00240087	0.05897583
ENOSF1	0.89740718	1.43688E-08	5.65472E-06	NRROS	-0.598932439	0.00241337	0.05912917
EPHB1	0.837348413	0.002359845	0.05826538	NUDCD1	-0.6602637	0.0002052	0.01025447
ETF1	0.67457154	0.001866114	0.049746318	NUDT3	-0.503747897	0.00066187	0.02372443
ETS2	1.01981433	0.001165902	0.035869521	OSBPL5	-3.070996131	6.3239E-06	0.00071719
EVC2	1.627088351	0.000178817	0.009254416	PCDH12	-0.686474855	0.00312702	0.07023702
EXT1	1.00192352	0.000201861	0.010172386	PGBD2	-0.570458881	0.00294913	0.06760813
EZH2	1.336922614	1.62195E-05	0.001509294	PHF23	-1.380271871	5.9293E-06	0.00068295
F13A1	0.63383494	0.004716543	0.092170551	PITPNM3	-0.829097362	0.00167111	0.0460163
FABP5	0.812318873	0.00109338	0.034192884	PLEKHA1	-0.587050823	0.00383796	0.08073393
FAM222A	1.05611047	0.000434432	0.017460481	POLN	-0.890821168	0.000111	0.00667194
FAM50B	1.101892982	0.000940578	0.030476002	POLR3E	-2.914866258	5.1177E-05	0.00368986
FAM76A	0.511708494	0.000833739	0.028073683	PPFIA4	-1.222001283	0.00061	0.02241849
FAM8A1	0.658883945	0.002362694	0.05826538	PRF1	-2.673125709	1.4929E-06	0.00022743
FANCA	1.227868245	2.87102E-08	9.51466E-06	PRKCQ	-0.750565463	0.00187929	0.04992936
FAS	0.665663106	0.001325888	0.039292039	PRUNE2	-2.321317083	7.1983E-05	0.00475438
FBXL14	1.969671571	2.88702E-07	6.05954E-05	PSMB8	-0.875512533	0.00216535	0.05468383
FBXO30	1.404870205	0.000220522	0.010876375	PTGER2	-2.418970701	8.2814E-05	0.0053391
FBXO33	0.772101398	0.000514644	0.019880626	PTPRO	-0.821679468	0.00128344	0.03854395
FBXO34	0.829515649	0.000485813	0.019158692	RAP1GAP2	-0.652909568	0.00467989	0.09179091
FBXO43	1.113519029	7.59306E-05	0.00495103	RCAN1	-1.286217258	1.6956E-09	7.8119E-07
FBXW7	0.567970676	0.000177677	0.009220631	RCSD1	-0.61003119	0.00072439	0.02543426
FGD4	0.675022597	0.003219727	0.071469605	RESF1	-0.788947715	0.00018936	0.00969357
FGD6	1.97385369	1.08646E-10	6.03625E-08	RHOC	-1.001860781	0.00069135	0.02450214
FGF2	0.836666105	0.00202654	0.052296922	RIN3	-1.550811918	7.8833E-08	2.1274E-05
FHOD3	1.790683104	6.14054E-05	0.004142671	RIPOR2	-0.870656762	5.4058E-05	0.00378203
FKBP14	1.628646451	3.82655E-06	0.000466346	RNF146	-0.53326666	0.00133103	0.03929204
FKBP4	2.365878341	3.48351E-11	2.26909E-08	RNF32	-0.519694482	0.00269238	0.06381793
FNBP1	0.749217715	5.51016E-05	0.003812705	RNF5	-2.416695623	5.3275E-05	0.00375934
FOSL1	2.45204843	1.41278E-06	0.000218749	RPAP1	-0.537457477	0.00011017	0.00665628
FOSL2	3.239063666	5.28869E-16	1.11004E-12	RPL26P6	-1.281685344	0.00063848	0.02319411
FST	1.340787549	0.000610011	0.022418494	RUSC2	-1.276204733	0.00065852	0.02364903
FSTL3	1.828795645	5.88549E-06	0.000682067	RYR2	-0.955480003	0.00063728	0.02319411
FXR1	0.857156656	3.96443E-07	7.72042E-05	SCARF2	-0.552472128	0.00520959	0.09733834
FYTTD1	0.789188234	0.000588213	0.02191586	SGF29	-0.500756968	0.00466219	0.09173396
FZD3	0.866550269	0.00067512	0.024016989	SGO1	-0.557587661	0.00106032	0.03338247
FZD6	0.617943454	0.002787596	0.065089843	SH2D2A	-0.771459145	0.00164863	0.04557895
G3BP2	0.567642371	0.002132099	0.054133544	SIGLEC17P	-0.500403878	0.00459685	0.09080759
GADD45A	1.469404231	4.56816E-06	0.000546155	SIGLEC7	-0.580086824	0.00305287	0.06931337
GAPDH	1.197448199	0.000187791	0.009639588	SKOR1	-1.387164147	6.5299E-06	0.00073422
GARS1	0.568287912	0.000336106	0.014495529	SLA	-0.709176291	0.00013418	0.00758874
GCAT	0.560278991	0.005353612	0.098957484	SLAMF8	-0.582956422	0.00273809	0.064298
GCH1	1.331994136	1.56528E-05	0.001471053	SLC27A2	-1.895451677	3.8368E-05	0.00298257
GEM	1.643243654	0.000156699	0.008486009	SLC66A1	-0.507538996	0.00153726	0.04334167
GFOD1	1.94377183	5.48526E-05	0.00380943	SLC6A16	-1.307392254	9.5975E-07	0.00016044
GLRX	1.178798512	0.00011789	0.007002971	SMBD1P	-0.629556686	0.00225163	0.05626091
GLS	0.653345773	0.002023354	0.052296922	SPRY2	-0.694604382	0.00330682	0.07297405
GNB3	2.228349887	6.57825E-05	0.004390923	SRBD1	-0.510388695	0.00125979	0.03801516
GNG2	1.811037494	7.60084E-05	0.00495103	SRC	-0.594709717	0.00419042	0.08548277
GNGT2	0.571752015	0.003787848	0.080222824	ST3GAL1	-0.569971371	0.00332103	0.07304804
GRHL1	0.653451186	0.004283825	0.086320118	STK38	-1.17299966	8.7795E-17	2.3692E-13
GTSE1	0.982680281	4.27389E-05	0.003191057	STON1	-1.185755703	0.00013925	0.00779255
GZF1	1.35527446	3.43544E-06	0.000435539	SUSD2	-1.255573851	2.3744E-05	0.00202042
H1-10	1.245547237	0.000269197	0.012555861	SYT2	-1.411163338	0.00032088	0.01419518
H1-2	3.565201129	5.00345E-07	9.35795E-05	TADA2A	-0.610540731	3.5562E-05	0.00281073
H1-3	4.929076911	2.39731E-06	0.000337455	TAF4	-0.938582212	0.00017507	0.00913558
H1-4	5.915534351	4.7488E-11	2.8937E-08	TAF5LP1	-0.60034875	0.00369993	0.07933218
H1-5	4.284334075	8.77282E-06	0.000946963	TANGO2	-0.58532399	0.0018888	0.04998469
H2AC11	3.433983993	7.01965E-08	1.95002E-05	TASOR	-0.783934891	0.00029726	0.01349838

H2AC13	1.648001358	0.00030016	0.01356466
H2AC14	1.452191398	0.000313849	0.013982547
H2AC16	2.531916262	2.91957E-05	0.002418886
H2AC17	4.182425708	1.67983E-06	0.000247906
H2AC20	2.322191832	6.48909E-05	0.004346769
H2AC21	0.533071044	0.004721511	0.092170551
H2AC6	2.364636733	4.13139E-05	0.003134217
H2AC7	2.132425164	9.57428E-05	0.006008576
H2AC8	3.693992245	1.27255E-06	0.000203716
H2AJ	0.719805783	0.003655859	0.078655105
H2BC10	0.844111772	0.001411224	0.040733878
H2BC11	2.943236345	1.62071E-06	0.000242979
H2BC12	0.968047289	0.001436654	0.041243758
H2BC13	1.454878317	0.00036443	0.015366938
H2BC14	1.427818615	0.00032985	0.014394246
H2BC15	1.902972188	9.11764E-06	0.000973063
H2BC17	1.190064294	0.000894923	0.029298273
H2BC18	2.11843707	2.54262E-05	0.002144203
H2BC19P	0.993849783	0.001398819	0.040612827
H2BC20P	0.795119463	0.002490659	0.060425093
H2BC21	2.805111115	2.24345E-08	7.84791E-06
H2BC4	2.857545709	2.4294E-06	0.000337455
H2BC5	2.425776188	2.93502E-06	0.000382362
H2BC7	2.346841488	8.12455E-05	0.005255919
H2BC8	3.19629879	1.14191E-05	0.001151909
H2BC9	1.487643857	0.000401669	0.016423237
H3-2	1.377192865	4.7591E-05	0.003457669
H3C1	2.246812733	4.31134E-05	0.003196233
H3C10	2.513261324	2.25965E-06	0.000322025
H3C12	2.006129981	0.000155881	0.008486009
H3C13	1.613944877	0.000299261	0.013556436
H3C2	0.970336192	0.000842184	0.028307557
H3C3	1.109250497	0.000666468	0.023822036
H3C4	3.089536829	2.21654E-05	0.001938446
H4C3	0.999143751	0.001370181	0.040190555
H4C5	0.65942697	0.004571884	0.090622136
HMCES	0.542403404	0.0019079	0.050335524
HMGA1	0.568110134	0.004043904	0.083122249
HMGB2	1.159561875	1.11434E-05	0.001137827
HMGB3	1.075678837	0.000227054	0.011140403
HMGCS1	0.538751827	0.002448788	0.059687235
HSP90AA1	2.748034157	1.15601E-15	1.81975E-12
HSP90AB1	1.891761444	4.01041E-10	2.10435E-07
HSP90AB2P	1.488458859	1.83799E-05	0.001653311
HSP90AB3P	0.721660271	0.003156591	0.070565681
HSPA1A	5.529776426	2.59141E-11	1.74828E-08
HSPA1B	2.424345853	7.72422E-06	0.000843413
HSPA1L	0.864073858	0.002171327	0.054688489
HSPA5	0.803444985	0.001119674	0.034787242
HSPA6	5.995798657	6.46833E-16	1.22187E-12
HSPA8	1.843542754	8.03395E-08	2.13748E-05
HSPA9	0.736936667	0.000120671	0.007145666
HSPB1	3.656639948	1.09948E-09	5.32545E-07
HSPD1	4.231105843	3.89378E-45	7.35535E-41
HSPE1	2.88614038	5.78099E-11	3.41259E-08
HSPH1	4.136434204	2.45969E-44	2.32318E-40
ICAM1	1.486188567	5.65146E-10	2.8853E-07
ICAM5	1.780453903	0.000233732	0.011350107
ID2	1.433233407	5.57088E-06	0.000649592
IER5	0.586477863	0.003785758	0.080222824
IFFO2	2.503005245	1.51867E-06	0.000229501
IFIT2	0.948050641	0.001412424	0.040733878
IFRD1	1.402444573	3.62054E-09	1.55436E-06
IGHG3	1.961345526	0.000144676	0.008014476
IGSF3	0.622709313	0.004891393	0.093982614
ILDR2	0.649041227	0.004795671	0.092817851
INA	1.741191826	0.000259665	0.012232117
TBC1D10B	-0.66115919	9.431E-06	0.00099526
TBX19	-0.765458509	0.00122493	0.03721869
TECPR2	-1.279157068	6.9499E-07	0.00012385
TECRP1	-1.24815306	0.00073553	0.0256822
TMLHE	-0.707743008	0.00030807	0.0138227
TOMM20P2	-0.692914829	0.00215	0.0543688
TRAPPC12	-0.509072635	6.2874E-05	0.00422667
TRBC1	-1.765681474	0.00012351	0.0072231
TRBC2	-1.568783706	8.9779E-06	0.00096359
TRDC	-0.600176283	0.00360985	0.07793147
TRGC1	-1.193643668	0.00088419	0.02900011
TRIM3	-1.352767433	9.672E-10	4.808E-07
TRIM56	-0.989939759	6.5268E-09	2.6802E-06
TTC30B	-0.657273448	0.00112926	0.03491295
TTC31	-0.524460955	1.3104E-05	0.00127835
TTC33	-0.606408123	0.00181341	0.04858905
UBQLN4	-0.869429168	5.7732E-07	0.00010692
UCP3	-1.244943158	6.2298E-07	0.00011208
UNC5CL	-0.958504236	0.00032221	0.01422101
WBP1L	-0.902650039	1.4251E-07	3.3513E-05
WSCD1	-1.266275133	0.00042065	0.01705176
ZBTB7B	-0.99262228	3.9922E-05	0.00308551
ZNF169	-0.886039948	5.3534E-05	0.00375934
ZNF316	-0.640737599	3.7717E-06	0.00046607
ZNF445	-0.603996874	0.0005872	0.02191586
ZNF747	-0.714474384	9.6667E-05	0.00604651
ZNF75D	-0.506267358	0.0016096	0.04475277
ZNF768	-0.509883842	0.00291506	0.06723506
ZNF876P	-0.54842652	0.00435392	0.0873095

INSIG1	0.939539759	0.000161897	0.008620878
IQGAP3	3.731946192	4.97712E-13	5.53046E-10
ISCA1	0.96745121	0.000275892	0.012742317
ISL2	0.803950007	0.001468734	0.041909952
ITGB4	0.578254925	0.00474821	0.092277459
JMJD1C	0.617202996	0.001803942	0.04856098
JMY	1.291723752	0.00058642	0.02191586
JOSD1	1.02035202	2.35852E-05	0.002015951
JPT1	0.991997598	0.000399791	0.016381883
KANK2	1.133848941	6.94376E-05	0.004602371
KBTBD6	0.574617319	0.003943804	0.081597434
KCNK12	0.665841591	0.004472935	0.089291537
KCTD12	2.073938375	3.23206E-08	1.01756E-05
KDF1	1.321836864	0.000645467	0.023268849
KIAA0895	0.842707702	0.000641002	0.023196415
KIF11	0.521445782	0.002737999	0.064297999
KIF14	0.827139786	0.000869677	0.028770916
KIF15	0.512145307	0.00180979	0.04856098
KIF18A	0.55886832	0.00130928	0.039009947
KIF18B	0.89383334	0.000414222	0.016827232
KIF23	1.27112388	2.9961E-13	3.53727E-10
KIF4A	1.231832655	0.000157951	0.008492978
KIF7	1.720990346	0.00019639	0.009945865
KIFC1	1.928715219	3.30109E-06	0.000424201
KLF10	2.21487412	2.87287E-06	0.000376865
KLF4	2.489229189	1.69785E-05	0.001564506
KLF5	2.791112839	1.64536E-06	0.000244731
KLF6	0.693666017	0.001739534	0.047485268
KLHL25	1.138187333	2.97954E-05	0.002447106
KPNA2	1.152820583	5.85841E-05	0.003995142
LAMP3	1.009505142	0.00095397	0.030699298
LAP3	0.74445646	0.000174161	0.009113297
LCK	0.51294257	0.004476394	0.089291537
LCP1	1.147541635	5.84267E-05	0.003995142
LDHA	0.888905713	0.000332996	0.014394246
LDHAP4	1.664607762	0.000244932	0.011713309
LDLRAD4	1.706066151	3.1815E-07	6.46221E-05
LEF1	2.190456946	1.1933E-08	4.79605E-06
LGALS3	0.760961973	0.001801445	0.04856098
LIMA1	0.742823395	0.002567638	0.061865669
LINC00643	0.723463365	0.002076907	0.053233063
LMNB1	0.676335023	0.001014979	0.032061784
LMO2	0.915341939	0.001918244	0.050537826
LONRF1	0.870010955	2.24683E-05	0.001946906
LRRC8C	0.846261526	0.000677151	0.024043943
LTBP1	1.521844838	0.000327949	0.014394246
MAFF	1.196720746	9.21957E-05	0.005824669
MAGEH1	0.621163104	0.004202862	0.085551784
MALLP2	2.548690448	1.7674E-05	0.001605102
MAP1LC3B	1.501857953	2.38045E-12	2.36667E-09
MAP1LC3B2	1.139945436	6.78097E-05	0.004510298
MAP3K8	1.881453547	1.14274E-07	2.76747E-05
MAPK1IP1L	0.608020171	0.000627107	0.022868835
MAPRE2	1.835638102	4.86622E-06	0.000578132
MAST4	1.065107605	1.71128E-05	0.001566099
MAT2A	0.506464678	0.000397196	0.016310949
MBD4	0.539062282	2.76484E-07	5.86828E-05
MBIP	0.669606211	0.000348711	0.014903062
MBNL2	1.647083549	1.42866E-05	0.001363003
MBOAT7	0.939710618	6.16565E-06	0.000705874
MCAM	1.608386435	2.2673E-06	0.000322025
MCL1	1.638063793	8.72063E-08	2.27595E-05
MDM2	1.138252213	3.79497E-06	0.00046607
MED26	0.533070865	0.003426238	0.074996102
MEF2B	1.320029299	0.000413949	0.016827232
MEIS2	1.642957259	4.63506E-05	0.003420165
MELTF	0.839012286	0.001451142	0.041533446

MFSD2A	0.592856585	0.001843421	0.049323272
MGAT3	0.873161696	0.00050992	0.019779026
MIDN	1.450678072	2.5863E-07	5.55174E-05
MKI67	1.669562923	5.50971E-06	0.00064645
MKNK2	1.109398095	1.7843E-09	8.0251E-07
MLLT3	0.797382705	0.000874341	0.02885378
MND1	2.29407867	1.41279E-05	0.0013547
MNS1	0.644136171	0.002613081	0.062403423
MOB3A	0.586219142	0.002645903	0.063107462
MREG	0.801810532	0.00057389	0.021552265
MRPL18	2.214398936	8.99124E-08	2.2952E-05
MT1F	6.168077003	4.38004E-12	3.93995E-09
MT1G	8.295649083	3.38787E-07	6.80818E-05
MT1H	2.472101454	9.91193E-05	0.006138895
MT1X	8.160375988	2.99599E-16	7.07429E-13
MT2A	8.315963956	1.79263E-11	1.30241E-08
MXD1	0.624741916	0.00199737	0.052041593
MYBL1	1.358103278	0.000195832	0.009944282
MYBL2	1.941742177	1.66833E-05	0.001544844
MYLIP	0.576786218	0.00374488	0.079842876
N4BP1	0.510991562	0.000845815	0.028328799
NAALAD2	2.028513069	4.01819E-05	0.003085515
NAB1	0.670839944	0.000645431	0.023268849
NABP1	0.518955803	0.001240491	0.037492596
NAMPT	1.866619626	3.13758E-05	0.002565752
NAMPTP1	1.898833643	4.17408E-05	0.003144612
NCAPG	1.543887723	4.42547E-07	8.53031E-05
NDRG1	1.221351033	1.31287E-05	0.001278353
NECTIN1	1.658706728	0.000257914	0.012180007
NET1	1.374225359	1.07436E-10	6.03625E-08
NFKB1	0.802656196	0.001306527	0.039005431
NINJ1	2.029730024	4.82516E-05	0.003492229
NKRF	1.477426379	2.23102E-08	7.84791E-06
NKX3-1	2.297625969	8.11098E-07	0.000138339
NOCT	1.370018182	2.05125E-05	0.001810656
NOD2	2.009128838	2.005E-05	0.00177814
NOP58	1.257663378	4.25813E-08	1.27676E-05
NR4A1	0.646416867	0.002923472	0.067346812
NR4A3	2.678727397	3.05847E-07	6.34885E-05
NRARP	2.165643935	3.19477E-05	0.002581942
NRBF2	0.557015493	0.000818725	0.027716331
NREP	0.6633283	0.000993509	0.031541836
NUDC	1.069155757	0.000165074	0.008734562
NUDT4	1.191221426	4.19504E-05	0.003144612
NUDT4B	0.797240646	0.001235157	0.037451231
NUF2	0.851300431	0.000426855	0.017229248
NUP58	1.222188933	0.000205168	0.010254474
NUSAP1	1.541054405	5.92093E-08	1.72832E-05
NXT2	0.622874351	0.002347196	0.058194959
ODC1	1.112361984	4.05522E-05	0.00310134
OSR2	2.279210919	1.86011E-07	4.12166E-05
OTULIN	1.143328029	0.000125143	0.007273696
OTULINL	1.380184861	1.53291E-05	0.001447829
OXCT2	0.72236239	0.003639597	0.078394522
OXSR1	0.554168933	0.002521799	0.061072788
P4HA2	1.478338871	9.82181E-05	0.006103095
PAK6	1.154751474	0.000616323	0.022606505
PAQR3	0.675188521	0.0023619	0.05826538
PARD6G	1.934112091	1.99862E-05	0.00177814
PARM1	0.593318868	0.004971302	0.094952367
PBK	1.58250337	5.27915E-05	0.003748989
PCGF5	0.76139326	0.000204291	0.010254474
PDIA5	0.83888479	0.000105929	0.006434055
PDIA6	0.510320889	0.001095114	0.034192884
PEG10	0.614283599	0.005415665	0.099225908
PER2	0.841151264	0.000232636	0.011328201
PEX5	1.047889941	1.00791E-05	0.001046121

PFKFB3	0.928925199	0.000531447	0.020280888
PGK1	0.780262897	0.00038887	0.016120216
PIGA	0.643496418	0.002707806	0.064013301
PIK3CG	0.924963385	0.000967745	0.030931809
PIM1	0.617412006	0.0046843	0.091790909
PIM3	0.869756561	0.00150226	0.042545252
PJVK	0.591415238	0.003065394	0.069435596
PKMYT1	1.593007986	3.57864E-05	0.00281669
PLB1	0.947134169	0.001690436	0.046480829
PLEK	0.625898669	0.001991992	0.051973394
PLEKHG2	0.892220501	0.000497272	0.019448189
PLEKHG7	0.612371033	0.004989121	0.095100391
PLK1	1.017803997	3.73899E-06	0.00046607
PMAIP1	1.312951008	0.00020194	0.010172386
POGLUT2	0.65186963	0.003823067	0.080600148
POLB	1.041096018	3.48874E-05	0.002769002
POU4F1	1.649775942	0.000302232	0.013625668
PPDPF	1.190471021	3.33637E-06	0.000425838
PPM1N	0.539602838	0.004286303	0.086320118
PPP1CC	0.714263404	0.000272854	0.01266389
PRDM1	1.254021157	0.000307304	0.013821347
PRDM2	1.01027118	1.23816E-06	0.000199905
PRKRA	0.503617604	0.001033142	0.032581062
PRMT9	1.249258488	9.85628E-08	2.48247E-05
PRNP	1.312859956	1.1349E-05	0.001151909
PRXL2C	0.774633849	2.30726E-05	0.001990145
PSMD10	0.579428872	0.000218744	0.01081694
PTEN	0.547143779	0.002229032	0.055772053
PTGER4	1.074671878	0.000775017	0.026582489
PTP4A1	0.910477923	0.00084818	0.028357736
PTTG1	0.578481102	0.005283248	0.098325674
RAB11FIP1	0.691672915	0.002945724	0.067608134
RAB1A	0.513151516	0.000620857	0.0226847
RALA	0.85264806	0.000264708	0.012377046
RALGDS	0.985908351	8.97349E-05	0.00572666
RANBP2	0.892970567	0.000471681	0.018797593
RAPGEF5	1.410312262	9.09861E-05	0.005786964
RASD1	0.687431728	0.002610128	0.062403423
RASL11A	0.774521597	0.002149228	0.054368802
RASSF5	0.718571023	0.000310757	0.01391042
RBM14	1.23697067	6.45747E-08	1.82062E-05
RELT	0.534894425	0.00138556	0.040427888
RFTN1	0.539071987	0.000743843	0.025876962
RGCC	0.604273992	0.003043476	0.069266578
RGS16	2.08645958	2.77466E-08	9.43425E-06
RGS8	6.891156617	2.08725E-06	0.000300978
RGS9	1.251588331	6.03474E-07	0.000110143
RHEB	0.609137516	0.001376003	0.040298753
RIPK2	0.822384572	0.000561191	0.021244269
RND1	0.698729038	0.003106988	0.069953523
RNMT	0.842868818	8.55547E-05	0.0054784
RO60	1.115830593	0.000143507	0.007973092
RPGR	0.85322511	0.000362139	0.015366938
RPL10P19	1.183444305	0.000830087	0.028000625
RPS27L	0.821094909	0.000777988	0.026623527
RRAD	0.712291778	0.002442593	0.059613145
RRAS2	0.504874463	0.004194946	0.085482777
RRP12	0.526916339	0.002010571	0.052241662
S100Z	1.590853017	0.000166149	0.00876691
S1PR2	1.079595422	0.000498768	0.019466375
SAMSN1	1.373040805	8.24499E-06	0.000895102
SAPCD2	1.445769229	1.87646E-07	4.12166E-05
SAR1A	0.548819244	0.001399627	0.040612827
SARAF	0.614533485	0.000155075	0.008486009
SDC4	0.835788313	0.002018539	0.052296922
SEC61B	0.633973635	0.003281862	0.072508041
SEL1L3	0.806319874	0.000393104	0.016213378

SEPTIN6	0.871170858	5.74523E-05	0.00394645
SERPINB9	0.525484276	0.003574231	0.077339319
SERTAD2	0.706320755	0.000181861	0.009360621
SETD7	0.566332407	0.002491855	0.060425093
SGK1	0.808379674	0.001426719	0.041081331
SH2B3	0.521685562	0.002148219	0.054368802
SH3PXD2A	1.204396354	2.77802E-05	0.002321986
SHQ1	0.569795166	0.001316273	0.039156524
SIAH2	1.232219286	2.58533E-05	0.002170528
SKA1	6.849988131	1.14054E-07	2.76747E-05
SKA3	1.026221897	0.000156957	0.008486009
SLBP	1.462813031	1.53767E-08	5.92788E-06
SLC12A7	0.819157177	0.001616708	0.044845237
SLC16A1	0.853390984	0.000757854	0.026219512
SLC16A6	0.840428443	0.000351574	0.01499149
SLC16A6P1	0.579179904	0.004518906	0.089911214
SLC1A4	0.6526909	0.000250803	0.011933668
SLC20A1	1.000631469	3.7422E-05	0.002921084
SLC25A4	0.658326802	0.002454123	0.059740178
SLC30A1	4.632630368	1.64492E-08	6.21451E-06
SLC5A3	1.241506642	0.000570489	0.021510049
SLC7A5	0.782544979	0.002752044	0.064339255
SLC9A1	0.51218115	0.000519589	0.020030696
SOCS3	2.086980898	1.215E-11	9.5631E-09
SOD1	0.777062394	0.001099039	0.034209394
SOX4	1.722004296	7.15891E-06	0.000795481
SPAG5	0.559021249	0.001556633	0.043731506
SPART	1.393386429	6.00913E-05	0.004083185
SPATA2	0.505859132	0.003722754	0.079731095
SPNS3	1.073433666	0.001217004	0.037166953
SPR	2.05108319	0.000125736	0.007285751
SPRED2	1.358398468	4.69966E-05	0.003427664
SPSB1	1.045456264	0.000668995	0.023843985
SQLE	0.55415685	0.001563422	0.043731506
SREBF1	0.691174754	0.002026143	0.052296922
SRGN	2.187706103	1.80348E-07	4.05569E-05
ST7	0.686350007	0.000396124	0.016302351
ST8SIA4	0.904417527	0.000602381	0.022224575
STAT4	1.260563115	1.37252E-06	0.000214273
STIP1	1.895931553	3.09422E-14	4.49614E-11
STPG3	0.978717041	0.001464822	0.041861554
STRAP	0.803305067	0.000312826	0.013969946
STX11	1.079415188	0.000798855	0.027132055
SVIL2P	0.812425363	0.002608067	0.062403423
SYAP1	1.78751428	1.07332E-21	6.75833E-18
SYBU	2.685227339	2.02853E-06	0.00029476
TAF9	0.656114686	0.002125585	0.054040773
TAF9B	0.944346775	9.71498E-05	0.006056636
TCAF2	0.931466937	0.000238399	0.011488176
TCAF2P1	0.592816619	0.004582391	0.090640173
TCEAL8	0.789679507	0.000560794	0.021244269
TCEAL9	1.166117716	0.000952926	0.030699298
TCP1	1.712224957	8.02576E-07	0.000138339
TEDC2	1.498009924	5.19096E-05	0.003714289
TENT4A	1.233692976	1.71616E-05	0.001566099
TESPA1	0.825621313	0.000377789	0.015753715
TEX9	1.034484546	0.000111355	0.006671936
TFRC	0.537562635	0.005060086	0.096065345
TICRR	0.93609822	0.000159317	0.008525504
TIPARP	1.051305669	0.000331019	0.014394246
TK1	0.797713274	0.000508562	0.019766962
TMC5	0.621466625	0.005100742	0.096607192
TMEM108	2.001780978	1.27835E-05	0.001264292
TMEM123	1.156966744	0.000124578	0.007263176
TMEM131L	0.621146315	0.000852533	0.028452904
TMIGD2	0.568827051	0.005486172	0.099936148
TNF	1.583076184	0.00035781	0.015223028

TNFAIP3	1.719525474	7.45447E-05	0.004906443
TNFRSF10B	0.806075784	0.000788508	0.026934742
TNFRSF11A	2.625547365	1.49838E-07	3.45175E-05
TNFRSF17	0.877582005	0.002105392	0.053671862
TNFSF9	0.675300553	0.00395863	0.081737551
TOB2	1.66024036	8.24373E-16	1.41567E-12
TOP1	0.650886681	0.000513319	0.019870077
TOP2A	0.587180825	0.000162469	0.008620878
TP53BP2	0.574386259	0.003831143	0.080680369
TPST1	1.473956878	6.34042E-06	0.000717189
TPX2	0.969993785	0.000131574	0.007486238
TRA2A	0.530291008	0.00015826	0.008492978
TRAM2	0.722372783	0.00274794	0.064339255
TRERF1	0.830512627	0.001902032	0.050250887
TRIM26	0.589476481	0.003866059	0.080964367
TRIM69	0.501704525	0.005447168	0.099637603
TSPYL4	0.578565115	0.003444363	0.075305567
TUBA1C	0.64052897	0.00170089	0.046587263
TUBA4A	0.615380618	0.002805762	0.065433145
TUBB	19.29248712	8.79536E-08	2.27595E-05
TUBB2A	1.246083756	0.000248303	0.011844541
TUBGCP5	0.592544008	0.002272499	0.056707414
TXNDC16	1.21456147	3.19838E-05	0.002581942
TXNDC5	1.359267401	4.19423E-05	0.003144612
TYMS	0.887728067	0.00128754	0.038605752
UAP1	0.518884396	0.005021637	0.095527412
UBB	0.981947255	0.000487995	0.019204628
UBC	0.971853124	0.000347353	0.014903062
UBE2A	0.682764689	0.000135192	0.007623217
UBE2C	0.645477988	0.00325467	0.071991475
UBE2H	0.708984523	0.000331807	0.014394246
UBQLN1	0.674850792	0.000791882	0.026960197
UFSP2	0.809952568	0.000264535	0.012377046
UHRF1	0.640136956	0.00196755	0.051406677
ULBP1	0.687387843	0.004061623	0.083395706
ULBP2	2.200174471	6.1178E-05	0.004142122
USP2	0.726220301	0.003189475	0.070881402
USP32P1	1.681884594	5.16149E-06	0.000609378
USP36	0.655660636	0.000600201	0.022187454
USP53	0.780885899	0.001073419	0.033738575
USPL1	0.837713093	0.000172974	0.009076329
VASN	0.712663584	0.001182165	0.036310722
VDAC2	0.51127097	0.002074065	0.053232449
VDR	2.307742653	7.68428E-06	0.000843413
VIM	0.560731953	0.003759364	0.080061322
WDR61	1.026362605	0.000728468	0.025482896
WNK2	2.279432364	3.23037E-05	0.002596666
XBP1	0.822928046	0.00212273	0.054040773
ZC3H12A	0.541912834	0.002582975	0.06199797
ZC3HAV1	1.156501006	1.29013E-11	9.74822E-09
ZCCHC24	0.774736454	0.00098357	0.031278843
ZFAND2A	1.630037436	1.33979E-06	0.000210905
ZFAND5	0.679480243	0.000654954	0.023565873
ZFAT	0.507845563	0.003876661	0.081028082
ZFP36	0.763911279	0.001934237	0.050746866
ZNF10	2.007964602	1.00777E-07	2.50485E-05
ZNF215	0.716056464	0.001796437	0.048553547
ZNF385B	2.583930758	1.82435E-05	0.001648895
ZNF844	0.745661644	0.000111964	0.006671936
ZWINT	0.831859905	0.001754546	0.047757018
